# Spatially diffuse cAMP signalling with oppositely biased GLP-1 receptor agonists in β-cells despite differences in receptor localisation

**DOI:** 10.1016/j.molmet.2025.102304

**Published:** 2025-12-12

**Authors:** Shiqian Chen, Carolina B. Lobato, Carissa Wong, Yusman Manchanda, Katrina Viloria, Iona Davies, Daniel B. Andersen, Julia Ast, Kyle W. Sloop, David J. Hodson, Johannes Broichhagen, Steve Bloom, Jens J. Holst, Tricia Tan, Alejandra Tomas, Ben Jones

**Affiliations:** 1 Section of Endocrinology, Department of Metabolism, Digestion and Reproduction, Imperial College London, London W12 0NN, UK; 2 Department of Biomedical Sciences, Faculty of Health and Medical Sciences, University of Copenhagen, Copenhagen, Denmark; 3 Section of Endocrinology, Department of Medicine, Copenhagen University Hospital – Amager and Hvidovre, Hvidovre, Denmark; 4 Section of Cell Biology and Functional Genomics, Department of Metabolism, Digestion and Reproduction, Imperial College London, London W12 0NN, UK; 5 Oxford Centre for Diabetes, Endocrinology and Metabolism (OCDEM), NIHR Oxford Biomedical Research Centre, Churchill Hospital, Radcliffe Department of Medicine, University of Oxford, Oxford, UK; 6 Institute of Metabolism and Systems Research (IMSR), and Centre of Membrane Proteins and Receptors (COMPARE), University of Birmingham, Birmingham, UK; 7 Novo Nordisk Research Centre Oxford, Innovation Building, Oxford, UK; 8 Diabetes, Obesity and Complications, Lilly Research Laboratories, Eli Lilly and Company, Indianapolis, IN 46285, USA; 9 Leibniz-Forschungsinstitut für Molekulare Pharmakologie (FMP), Berlin, Germany; 10 Department of Chemical Biology, Max Planck Institute for Medical Research, Heidelberg, Germany; 11 Novo Nordisk Foundation Center for Basic Metabolic Research, Faculty of Health and Medical Sciences, University of Copenhagen, Copenhagen, Denmark

**Keywords:** GLP-1, GPCR, Biased agonism, Obesity, Type 2 diabetes

## Abstract

Internalisation of G protein-coupled receptors (GPCRs) can contribute to altered cellular responses by directing signalling from non-canonical locations, such as endosomes. If signalling processes are locally constrained, active receptors in different subcellular locations could produce different downstream effects. This phenomenon may be relevant to the optimal targeting of the glucagon-like peptide-1 receptor (GLP-1R), a type 2 diabetes and obesity target GPCR for which several ligands with varying internalisation tendency have been discovered. To investigate, we compared the signalling localisation effects of two prototypical GLP-1RAs with opposite signal bias and effects on GLP-1R trafficking: exendin-asp3 (ExD3), a full agonist that drives rapid internalisation, and exendin-phe1 (ExF1), which shows much slower internalisation. After using bioorthogonal labelling and fluorescent agonist conjugates to verify the divergent trafficking patterns of ExF1 and ExD3 in β-cell lines and primary pancreatic islets, we used live cell biosensors to monitor signalling at different subcellular locations. This revealed that cAMP/PKA/ERK signalling in β-cells is in fact distributed widely across the cell over short- (<5 min) and medium-term (up to 60 min) stimulation at pharmacological (>10 pM) concentrations, with no major differences in signal localisation that could be linked to internalised *versus* cell surface-bound GLP-1R. Moreover, washout experiments highlighted that, whilst fast-internalising ExD3 shows much greater accumulation and binding to GLP-1R in endosomes than slow-internalising ExF1, it is a rather inefficient driver of both cAMP production in β-cells and insulin secretion from perfused rat pancreata. These data provide a greater understanding of the cellular effects of biased GLP-1R agonism.

## Highlights

• Oppositely biased GLP-1R agonists show distinct receptor internalisation responses.
• We confirm that GLP-1R continues to signal after internalisation.
• However, cAMP/PKA/ERK signalling is spatially diffuse irrespective of receptor location.
• Differences in receptor location did not influence short term pancreatic islet hormone secretion.

## 1 Introduction

The glucagon-like peptide-1 receptor (GLP-1R) is a class B1 G protein-coupled receptor (GPCR) that is well established as a treatment target for type 2 diabetes (T2D), obesity, and related metabolic diseases [1]. Activation of GLP-1R by peptide ligands such as exendin-4 and semaglutide potentiates insulin secretion from pancreatic β-cells and suppresses appetite through action at anorectic neurons [2]. The agonist-bound GLP-1R activates Gα subunits including Gα_s_ and Gα_q_ [3], leading to increases in cytosolic cyclic adenosine monophosphate (cAMP) and Ca^2+^ levels. It also recruits β-arrestins that terminate G protein signalling and, separately, might facilitate non-G protein-mediated signalling cascades [4]. Whilst traditional signalling readouts measure whole-cell responses, the rapid agonist-mediated endocytosis of the GLP-1R has generated much interest in the idea of spatially segregated signalling from the endosomal compartment [5,6], as described for many other GPCRs [[7], [8], [9], [10]]. Persistent activation of internalised receptors provides a means to extend signalling duration, but also to encode distinct response types due to proximity with different groups of downstream effectors at different locations. Highly localised GLP-1R signalling nanodomains at the plasma membrane have also been reported, which could also contribute to response diversity [[11], [12], [13]].

In this context, interest has emerged in GLP-1R agonist ligands (GLP-1RAs) showing distinct trafficking profiles, which could lead to altered signalling from intracellular locations [[14], [15], [16]], as has been extensively noted for other GPCRs [[17], [18], [19]]. Interestingly, despite the proposed importance of endosomal signalling, many studies have demonstrated that GLP-1RAs showing reduced GLP-1R endocytosis are in fact able to prolong glucose-stimulated insulin secretory responses through avoidance of target downregulation and preservation of signalling from the plasma membrane [[20], [21], [22]]. Indeed, it is postulated that the therapeutic performance in humans of two more recently developed GLP-1RAs – tirzepatide and orforglipron – depend partly on their avoidance of GLP-1R desensitisation or downregulation [[22], [23], [24], [25]]. On the other hand, others have attributed enhanced GLP-1R-mediated delivery to early endosomes of the GLP-1RA/GIPR antagonistic antibody AMG133 (maridebart cafraglutide or “MariTide”) as a driver of its powerful effects [6,26].

Although therapeutically insightful, direct comparisons of these clinical agents provide limited mechanistic insights into the consequences of altered GLP-1R trafficking and biased signalling due to differences in biodistribution, pharmacokinetics, receptor specificity (e.g. activity at GIPR as for tirzepatide and MariTide) and species specificity (e.g. orforglipron is inactive in rodent systems). Therefore, in this work we aimed to use two exemplar and oppositely biased GLP-1RAs – “exendin-phe1” (ExF1) and “exendin-asp3” (ExD3) – that are known to show highly divergent effects on GLP-1R internalisation [20]. Specifically, ExD3 leads to high levels of internalisation – even greater than the reference agonists GLP-1 and exendin-4 themselves - whereas ExF1 shows much reduced internalisation tendency. Due to their highly contrasting effects at the GLP-1R despite strong structural and pharmacokinetic similarity, ExF1, ExD3 and closely related derivatives are frequently used as tools to probe the effects of GLP-1R biased agonism in experimental studies [11,21,[27], [28], [29], [30], [31], [32]], meaning that a deep understanding of their cellular effects will be valuable to the community. As much of the earlier work has been conducted using heterologous cellular systems which could introduce artefacts from high levels of receptor expression, we aimed in here to focus on ExF1 and ExD3-specific patterns of signalling in pancreatic β-cell lines and pancreatic islets that express GLP-1R endogenously. The key question we have aimed to address is whether the differences in GLP-1R distribution produced by these ligands leads to differences in the magnitude, temporal dynamics and spatial localisation of cAMP signalling.

### 1.1 Marked differences in GLP-1R internalisation induced by ExF1 and ExD3

As tools to probe the importance of GLP-1R subcellular location, we made use of the N-terminally modified exendin-4 analogues ExF1 and ExD3, as previous work has shown that ExF1 leads to reduced acute GLP-1R internalisation *versus* ExD3 [20]. We first aimed to compare responses at human *versus* mouse GLP-1R, which is important given the common use of mice in metabolic research. We used human and mouse GLP-1R constructs (hGLP-1R and mGLP-1R, respectively) with an N-terminal SNAP-tag to allow surface-specific labelling with fluorescent dyes coupled to O^6^-benzylguanine. Both species of SNAP-tagged receptor, and an additional pair further modified with a C-terminal nanoluciferase for BRET studies, were expressed at the cell surface in adherent HEK293 (AD293) cells and produced appropriate cAMP responses to agonist stimulation (Supplementary Fig. 1A and 1B). The SNAP-mGLP-1R constructs showed higher surface expression than the human equivalent, as measured by the SNAP-labelling signal, and showed higher cAMP potency for both ligands. ΔLogE_max_/EC_50_ analysis [33] indicated that the greater cAMP potency of ExF1 at SNAP-mGLP1R was partly intrinsic to the receptor and not entirely explained by differences in surface expression (Supplementary Fig. 1C).

**Figure 1:**
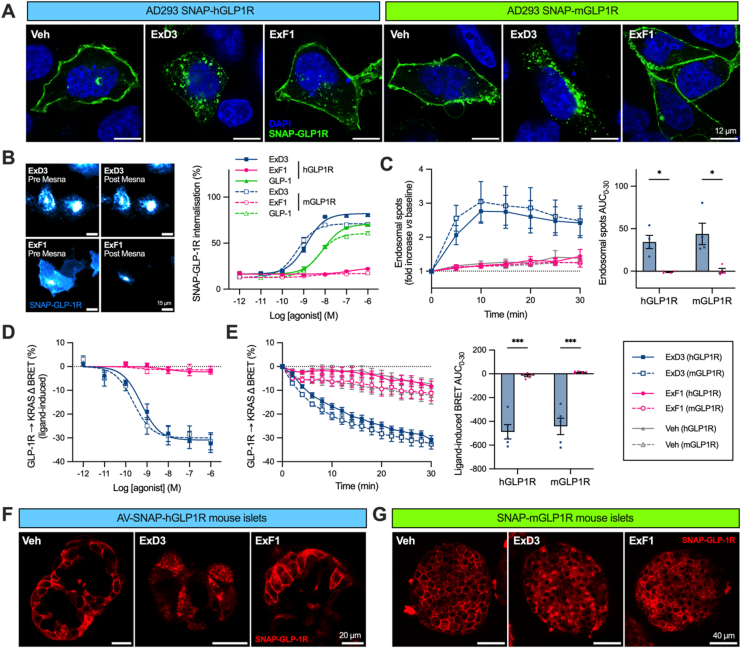
**Human and mouse GLP-1R internalisation responses to biased agonist stimulation.** (**A**) Agonist-induced GLP-1R internalisation in AD293 cells transiently expressing SNAP-hGLP-1R or SNAP-mGLP-1R and labelled with SNAP-Surface-488 prior to 30-minute 100 nM stimulation, representative of *n* = 3, scale bar = 12 μm. (**B**) Agonist-induced GLP-1R internalisation in AD293 cells quantified using Mesna assay at 30 min, *n* = 5, 3-parameter logistic fit, with representative images showing effects at SNAP-hGLP-1R, scale bar = 15 um. (**C**) Time course showing appearance of SNAP-GLP-1R in endosomal puncta in response to 100 nM agonist, *n* = 4, statistical comparison by two-way matched ANOVA with Sidak's test from vehicle-subtracted AUC. (**D**) Agonist-induced GLP-1R internalisation quantified at 30 min using bystander BRET between GLP-1R-Nluc and KRAS-venus, *n* = 6, 3-parameter logistic fit with subtraction of fitted baseline response. (**E**) 100 nM agonist-induced GLP-1R internalisation time-course via bystander BRET, *n* = 5, with comparison by two-way matched ANOVA with Sidak's test from vehicle-subtracted AUC. (**F**) Representative images of intact mouse pancreatic islets transduced with adenovirally encoded AV-SNAP-hGLP1R, treated with 100 nM agonist for 30 min after labelling with SNAP-Surface-549, *n* = 3. (**G**) Islets from SNAP-GLP-1R mice labelled with BG-Sulfo646 before treatment with 100 nM agonist for 30 min, *n* = 3. ∗p < 0.05, ∗∗∗p < 0.001 by indicated statistical test. Data represented as mean ± SEM.

As expected, there were obvious differences in the ability of ExF1 and ExD3 to drive SNAP-GLP-1R internalisation in AD293 cells (Figure 1A). To quantify this, we used a reversible labelling assay based on cleavable dyes [20,34]; this indicated that ∼20% of GLP-1R from both species is constitutively internalised over 30 min, and that this is greatly enhanced by ExD3 treatment but only minimally by ExF1 (Figure 1B). Internalisation potency for ExD3 was mildly increased at SNAP-mGLP-1R compared to SNAP-hGLP-1R (EC_50_ = 0.5 *versus* 1.2 nM), but with a small reduction in maximum response (E_max_ = 73% *versus* 82%). ExD3 was notably more potent than native GLP-1(7–36)NH_2_, as well as achieving a moderately higher maximal response. Real time imaging of SNAP-GLP-1R redistribution into endosomal puncta highlighted the rapid kinetics of internalisation with ExD3 (Figure 1C). These results were corroborated by measuring the disappearance of nanoluciferase-tagged SNAP-GLP-1R from the plasma membrane (KRAS-Venus marker) using bystander BRET, which confirmed much more GLP-1R internalisation with ExD3 (Figure 1D, E), accompanied by its accumulation in early endosomes (Rab5-Venus marker; Supplementary Fig. 1D).

To extend these observations to the native environment of the GLP-1R, we imaged agonist responses in intact pancreatic islets transduced with adenovirus-encoded SNAP-hGLP-1R, and from a recently developed knock-in mouse model that expresses SNAP-mGLP-1R from the endogenous locus [13]. The main observation from these experiments was clearly greater GLP-1R internalisation with ExD3 than ExF1 (Figure 1F, G), matching the cell line observations. We note again that a degree of constitutive GLP-1R internalisation was apparent from the images of vehicle- and ExF1-treated samples.

### 1.2 Fluorescent biased GLP-1RAs for studying responses with untagged GLP-1R

**Figure 2:**
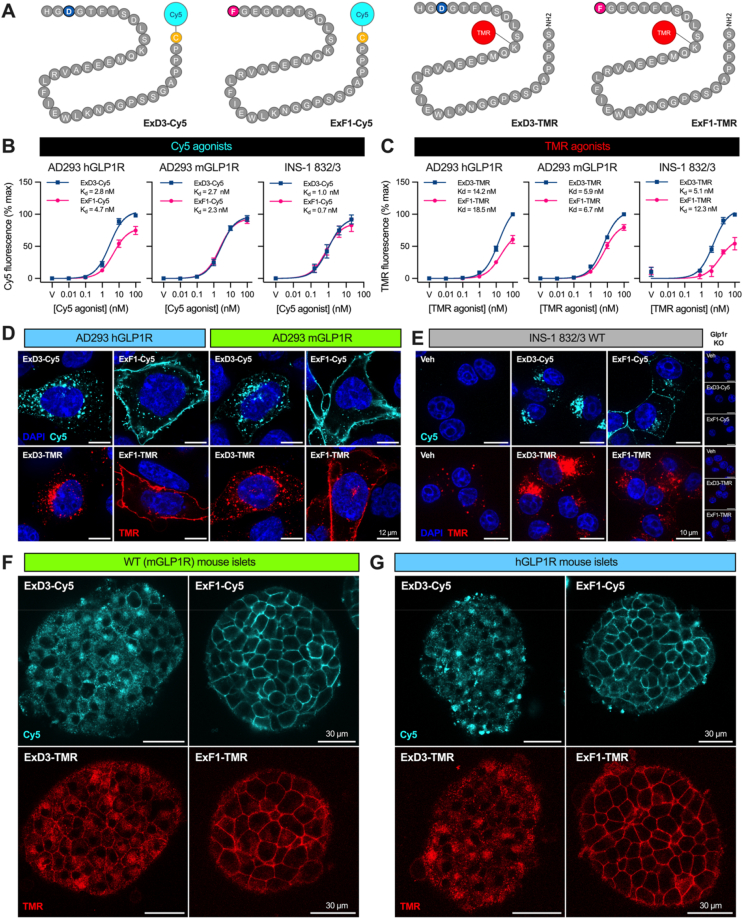
**Fluorescent conjugates for monitoring of biased agonist trafficking without receptor tagging.** (**A**) Cy5 and TMR fluorophore conjugation positions. (**B**) Quantification of bound Cy5-agonist fluorescence in hGLP-1R- or mGLP-1R-expressing AD293 cells, or wild-type INS-1 832/3 cells, after 30 min stimulation. For INS-1 832/3 cells, non-specific binding has been calculated from concurrently analysed INS-1 832/3 *Glp1r*^−/−^ cells and subtracted. Signal is normalised to the highest signal data point for each assay, *n* = 5–6. (**C**) As for (B) but using TMR-agonists, *n* = 4–5. (**D**) Cy5- (3 nM) or TMR-agonist (10 nM) distribution in AD293 cells expressing hGLP-1R or mGLP1R after 30-minute stimulation, scale bar = 12 μm, representative of *n* = 3 experiments. (**E**) Cy5- (3 nM) or TMR-agonist (10 nM) distribution in INS-1 832/3 cells after 30-minute stimulation, with images from *Glp1r*^*−/−*^ shown for comparison, scale bar = 12 μm, representative of *n* = 3 experiments. (**F**) Representative confocal images of intact pancreatic islets from wild-type C57Bl/6J mice treated with Cy5-or TMR-agonists (100 nM) for 30 min prior to imaging. (**G**) As for (F) but using humanised GLP-1R mice. Data represented as mean ± SEM.

To study GLP-1R internalisation without potential interference from receptor tagging, we also developed fluorescent analogues of ExF1 and ExD3, modified using Cy5 or tetramethylrhodamine (TMR) fluorophores (Figure 2A). By imaging the binding of fluorescent ligands to untagged GLP-1R expressed in AD293 cells, and INS-1 832/3 clonal rat β-cells which endogenously express GLP-1R [35], we established functional affinities in the low nanomolar range for each ligand (Figure 2B, C). However, the TMR conjugates showed approximately 5-fold lower affinity at the hGLP-1R, and 3-fold lower affinity at the mGLP-1R, compared to Cy5 equivalents in these experiments. Except for ExF1-Cy5 at the mGLP-1R, ExD3-based conjugates produced higher total binding, which is likely to reflect greater ligand accumulation in endosomes over time due to the greater internalisation tendency of ExD3. Despite the apparently lower binding affinity of the TMR conjugates, cAMP signalling potencies for both sets of fluorescent analogues were similar, and typically ∼3-fold lower than for non-modified ExD3 and ExF1. An exception was ExF1-Cy5, which showed similar potency (although a lower maximal response) to non-modified ExF1 at the mGLP-1R (Supplementary Fig. 2A and 2B).

Both sets of fluorescent agonists showed the expected pattern of internalisation with untagged GLP-1R, with ExD3 conjugates extensively internalised and ExF1 conjugates remaining mainly (but not exclusively) at the cell surface after 30 min of agonist exposure in GLP-1R-expressing AD293 cells (Figure 2D). All ligand conjugates were specific for GLP-1R as they only bound to cells expressing SNAP-GLP-1R in transiently transfected pools (Supplementary Fig. 2C and 2D). The Cy5-tagged ligands proved useful to visualise low levels of endogenously expressed GLP-1R in INS-1 832/3 cells, revealing the expected pattern of internalisation, with ExD3-Cy5 predominantly intracellular and ExF1-Cy5 still showing a substantial amount of plasma membrane localisation after 30 min (Figure 2E). The TMR-tagged ligands were less useful in this cell model due to spectral overlap with autofluorescent granules, but differences in uptake of ExF1-TMR and ExD3-TMR could still be discerned (Figure 2E).

Ligands with both fluorescent moieties produced strong signals in wild-type mouse islets, with ExD3 conjugates extensively internalised and ExF1 conjugates remaining more at the cell surface (Figure 2F). To study the hGLP-1R in an islet context we made use of mice in which the endogenous *Glp1r* sequence has been replaced with that of the hGLP-1R [36]. We noted that surface GLP-1R expression was somewhat reduced in humanised GLP-1R compared to wild-type mouse islets, as assessed by *in vitro* labelling of islets using exendin-4-Cy5 (Supplementary Fig. 2E); low expression of humanised GLP-1R in a mouse model with the same genetic design has been remarked on elsewhere [37,38]. Nevertheless, the subcellular distribution of fluorescent ExF1 and ExD3 conjugates was very similar in hGLP-1R mouse islets to that in wild-type mouse islets (Figure 2G).

Overall, these data demonstrate the marked but consistent differences in GLP-1R internalisation with ExF1 compared to ExD3 across different cellular backgrounds, including in pancreatic islets.

### 1.3 Understanding ligand–receptor interactions at the subcellular level using FRET

**Figure 3:**
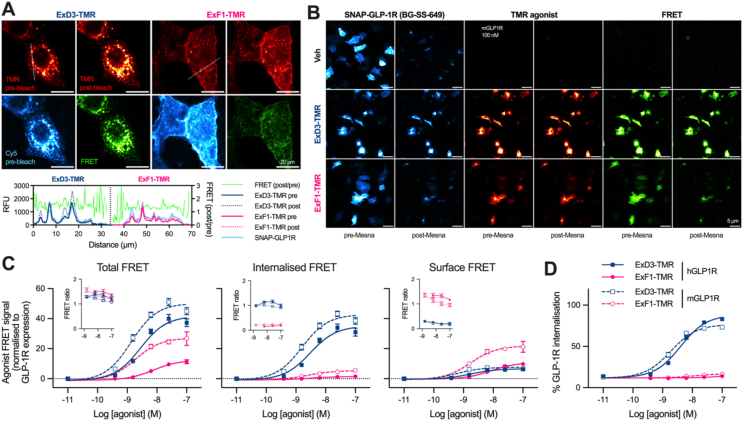
**FRET imaging of GLP-1R agonist binding within the cell.** (**A**) Representative images of AD293 cells expressing SNAP-mGLP1R, labelled with SNAP-Surface-AlexaFluor 647 and treated with 100 nM ExF1-TMR or ExD3-TMR for 30 min. Increased TMR-agonist fluorescence, shown by subtraction in the images and as a ratio (right y-axis) in the line scans, is apparent after photobleaching of the SNAP-probe and indicates binding of agonist to GLP-1R. Scale bars = 20 μm. (**B**) Representative images of AD293 cells expressing SNAP-mGLP1R, labelled with BG–SS–649 and treated with 100 nM ExF1-TMR or ExD3-TMR for 30 min, and imaged before and after Mesna treatment. Scale bar = 5 μm. (**C**) Quantification of TMR-agonist/GLP-1R agonist binding from (B), *n* = 5, with the pre-Mesna FRET signal representing “total FRET”, the post-Mesna signal “internalised FRET”, and the difference between the two “surface FRET”. The insets show the FRET ratio at each concentration, calculated by dividing the FRET signal by the direct TMR-agonist signal. (**D**) Quantification of GLP-1R internalisation in response to 30 min TMR-agonist treatment as in (B), *n* = 5 experiments. Data represented as mean ± SEM.

The assays above highlight agonist-specific differences in the acute distribution of both GLP-1R and its ligands, but do not indicate whether they remain bound after entering the endocytic pathway. Persistence of agonist/receptor complexes is an important factor controlling post-endocytic sorting and endosomal signalling [39]. To address this, we used FRET imaging to detect interaction between ExF1-TMR or ExD3-TMR (FRET donors) and GLP-1R labelled with a far-red SNAP-tag probe (FRET acceptor). We used TMR rather than Cy5 agonist conjugates for these experiments because the latter would require a near-infrared acceptor SNAP-probe, which is not commercially available. High resolution imaging of ExF1- and ExD3-TMR using acceptor photobleaching demonstrated FRET arising from both surface-bound and internalised ligand (Figure 3A).

By adapting the reversible SNAP-labelling approach from Figure 1B we developed a high throughput ratiometric FRET-based approach to separately quantify binding at the cell surface and from internal compartments. Because Mesna treatment in this experiment removes surface SNAP-GLP-1R fluorescence but does not remove direct TMR agonist signal (Supplementary Fig. 3A and 3B), FRET images captured before and after Mesna treatment allowed “total” and “internalised” receptor-bound fluorescent agonist to be separately quantified (Figure 3B), with the difference between the two indicating bound surface-bound ligand. In this context, we use “FRET signal” as a measure of the *amount* of bound agonist, and “FRET ratio” (i.e. expressing the FRET signal relative to the direct TMR agonist signal) to refer to the *proportion* of visualised agonist bound to receptor. Across the concentration range, the total FRET signal with ExD3-TMR was higher than that with ExF1-TMR at both human and mouse SNAP-GLP-1R, with the difference arising mainly from internalised ligand (Figure 3C). Indeed, with the SNAP-mGLP-1R, at concentrations greater than 3 nM there was more bound ExD3-TMR in the internalised compartment than the maximal binding achievable with high concentrations of ExF1-TMR. In contrast, there was more ExF1-TMR than ExD3-TMR recorded as bound to surface SNAP-GLP-1R, at least at higher agonist concentrations. Of note, despite normalising to differences in surface GLP-1R expression, both ligands showed higher maximal FRET signal at the SNAP-mGLP-1R compared to SNAP-hGLP-1R (Figure 3C). This could reflect species-specific differences in binding and receptor biology, but differences in FRET distance resulting from species-specific receptor conformations are also possible. Verifying the suitability of TMR conjugates, SNAP-GLP-1R internalisation quantification after 30 min stimulation from these experiments (Figure 3D) showed a very similar pattern to that seen with non-fluorescent ligands in Figure 1B.

These data highlight the higher level of intracellular ExD3 bound to GLP-1R compared to ExF1 after short term stimulations.

### 1.4 Agonist-specific GLP-1R internalisation differences are not associated with altered nuclear *versus* plasma membrane cAMP/PKA/ERK signalling in β-cells at steady state

**Figure 4:**
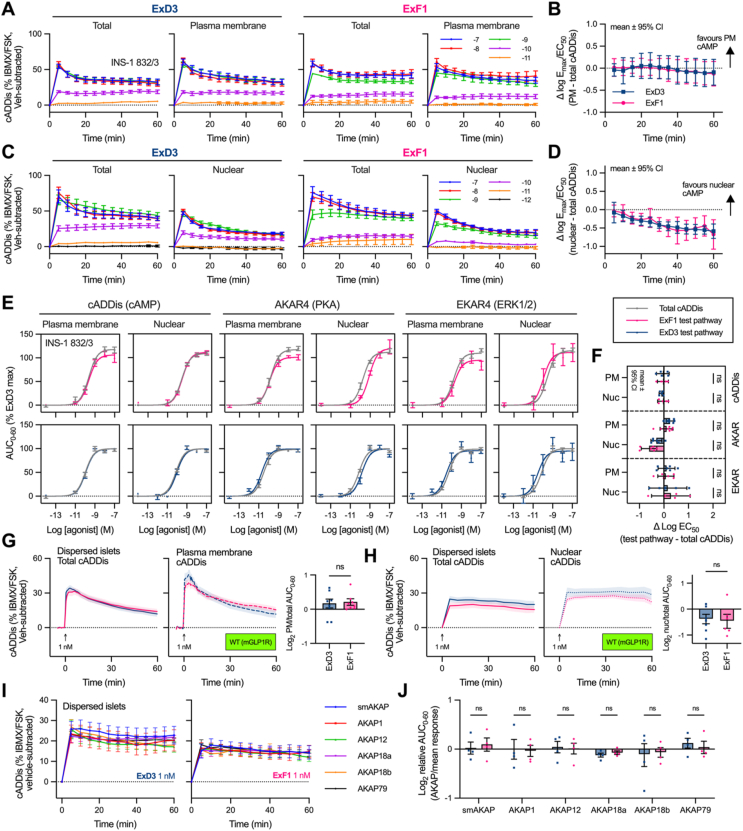
**Lack of ligand-specific cAMP signalling localisation with ExF1 and ExD3.** (**A**) Total and plasma membrane cADDis responses with normalisation to IBMX/FSK control and subtraction of vehicle response, *n* = 5. The legend indicates agonist concentration in log molar units. (**B**) Quantification of signal localisation by subtracting log E_max_/EC_50_ for each ligand/pathway using data from (A), with 95% confidence intervals. (**C**) As for (A) but nuclear and total cADDis. (**D**) As for (B)but using data from (C). (**E**) Targeted cADDis, AKAR4 and EKAR4 sensor responses in INS-1 832/3 cells, calculated from the full 60-minute stimulation period, with normalisation to the ExD3 maximum response in each assay repeat, *n* = 5–7. The whole-cell cADDis response from the same samples is shown in grey. (**F**) Quantification of signal localisation using data from (E), by subtracting pEC_50_ values for localised sensor *versus* whole-cell cADDis. Preferential signalling at the targeted sensor location is indicated by values over 0; 95% confidence intervals are shown, and ExF1 and ExD3 are compared by multiple t-tests with Holm-Sidak correction. (**G**) Total and plasma membrane cADDis responses in dispersed islets from C57Bl/6J mice stimulated with 1 nM agonist or vehicle, *n* = 8, with paired t-test to compare vehicle-subtracted AUC. (**H**) As for (G) but comparing total and nuclear cADDis responses, *n* = 7. (**I**) AKAP-localised cADDis responses in dispersed islets from wild-type C57Bl/6J mice, all tested in parallel in response to 60-min stimulation with 1 nM ExF1 or ExD3, *n* = 4. (**J**) Quantification of AKAP-specific signal localisation by expressing AUC from (I) relative to the mean AUC from all 6 sensors, with comparison by multiple paired t-tests with Holm-Sidak correction. Data represented as mean ± SEM unless indicated as 95% confidence intervals.

Several reports highlight the spatial specificity of GLP-1R signalling [5,12,16,40,41], so we wondered if the distinct trafficking profiles of ExF1 and ExD3 would lead to preferential signalling within distinct subcellular compartments. Specifically, we wondered whether the greater tendency of ExF1 to preserve GLP-1R at the cell surface would result in more plasma membrane-localised signalling and a failure to engage with broader signalling events deeper in the cell. To investigate, we used single wavelength cADDis cAMP sensors [42] targeted to spatially distinct locations for imaging-based measurements of real-time [cAMP] increases in INS-1 832/3 β-cells. We used this β-cell model because it expresses GLP-1R at endogenous levels, thereby avoiding artefacts from highly amplified cAMP responses seen in heterologous systems. Imaging was performed using a high content platform, allowing hundreds of cells to be monitored in parallel for increased statistical robustness. Using nucleus- or plasma membrane-targeted sensors alongside a non-targeted but spectrally distinct whole cell cADDis cAMP sensor, we were able to multiplex measurements to allow simultaneous recordings from the same cell population in response to 60-minute agonist stimulations across a full concentration range (Figure 4A–D; Supplementary Fig. 4A). Agonist responses fell within the dynamic range of the sensor established by adding forskolin/IBMX (Figure 4A, C). Concentration responses were constructed for each time-point, allowing comparison of signalling responses with each sensor by subtraction of logarithms (ΔLogE_max_/EC_50_). This analysis showed no difference in the location specificity of ExF1 *versus* ExD3 cAMP signalling, with plasma membrane responses closely mirroring those recorded from the whole cell (Figure 4B), and nuclear cAMP responses being somewhat reduced relative to the whole cell but with no difference between agonists (Figure 4D). No evidence of time-dependent differences in signalling location emerged throughout the 60-minute stimulation period.

We investigated further using membrane- and nucleus-localised CFP/YFP-based FRET-based protein kinase A (PKA) and ERK1/2 sensors [43,44] (Supplementary Fig. 4A), with multiplexed whole cell cADDis cAMP at a different wavelength used as a reference. In each case, integrated sensor responses measured over the 60-minute stimulation period showed little evidence of differential localisation of ExF1 and ExD3 signalling, as can be seen from the overlayed concentration response curves (Figure 4E) and EC_50_ comparisons (Figure 4F). Re-analysis of the cADDis cAMP data from Figure 4A–D using the 60-minute integration approach are also included for comparison. We did however note the nuclear PKA response with both agonists showed moderately reduced potency compared to whole cell cAMP, but again there was no significant difference between agonists. We also compared cAMP responses using cADDis sensors targeted to different compartments using different A-kinase-anchoring protein (AKAP) sequences, including within the plasma membrane (smAKAP, AKAP12, AKAP18a, AKAP18b, AKAP79) and outer mitochondrial membrane (AKAP1). Although we did not independently validate their localisation, it was apparent that all sensors yielded similar concentration responses for each agonist (Supplementary Fig. 4B and 4C). We repeated cADDis cAMP measurements in hGLP-1R- or mGLP-1R-expressing AD293 cells, which did not show strong evidence of agonist-specific signal localisation, although there was a non-significant trend towards greater plasma membrane signalling with ExF1 (Supplementary Fig. 4D–4G). Recordings using our *in house*-developed cAMP FRET sensor ^T^Epac^VV^ targeted to plasma membrane *versus* endosome [41,45] did not suggest that greater internalisation of ExD3 leads to higher concentrations of endosomal cAMP in INS-1 832/3 cells (Supplementary Fig. 4H and 4I) or in hGLP-1R- or mGLP-1R-expressing AD293 cells (Supplementary Fig. 4J and 4K).

We aimed to validate these findings in primary mouse islet cells. For these experiments we used 1 nM agonist, as FRET binding data with mGLP-1R presented in Figure 3C suggested that this concentration produces almost exclusively endosomal binding with ExD3 but only surface binding with ExF1 at the 30-minute time point. Despite the stark differences in receptor location under these conditions, cAMP responses measured using membrane-tethered or nucleus-localised *versus* whole cell cADDis (Figure 4G, H), or with different AKAP-directed cADDis (Figure 4I, J), were very similar between agonists, again suggesting that cAMP production with ExF1 or ExD3 in dispersed islet cells is not spatially restricted to its site of generation.

Overall, our results suggest the pronounced differences in GLP-1R location driven by oppositely biased agonists are not accompanied by segregation of cAMP production in β-cells at the whole organelle level over a 60-minute period.

### 1.5 GLP-1R-induced cAMP accumulation is rapidly observed at the cell nucleus

**Figure 5:**
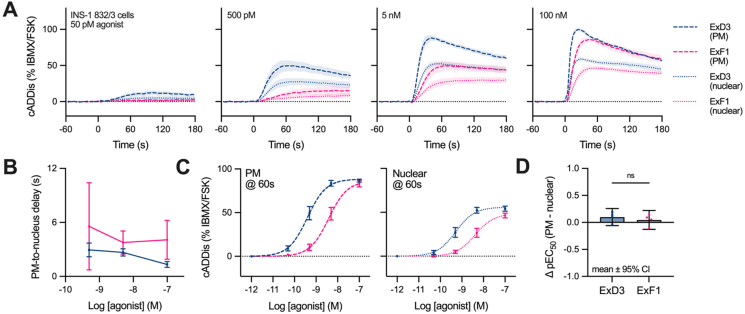
**Rapid increases in nuclear cAMP are detected following GLP-1R stimulation at the plasma membrane.** (**A**) Plasma membrane (PM) and nuclear cAMP kinetic measurements in INS-1 832/3 cells in response to indicated concentration of agonist, *n* = 4. (**B**) Delay between plasma membrane and nuclear cAMP estimated by comparing the difference between time-to-half max for each sensor. (**C**) Sensor responses at 60 s at several concentrations, with 3-parameter logistic fit. (**D**) Quantification of signal localisation using data from (C), by subtracting pEC_50_ values for PM from nuclear cADDis. 95% confidence intervals are shown, and ExF1 and ExD3 are compared paired t-test. Data represented as mean ± SEM unless indicated.

Experiments presented in Figure 4 aimed to characterise GLP-1R signalling over a 60-minute agonist exposure period. To test whether the kinetic resolution of experiments had missed early differences in cAMP signal localisation, we performed additional experiments focussed on short-term responses in INS-1 832/3 β-cells, using plasma membrane- and nucleus-targeted cAMP biosensors to maximise distance between locations. Simultaneous recordings from these spectrally distinct sensors increases the validity of kinetic comparisons by avoiding artefacts e.g. due to delayed sample mixing following agonist addition. Across a range of agonist concentrations, cAMP accumulation at the plasma membrane preceded that in the nucleus by a few seconds, and this delay was similar with ExF1 and ExD3 across a range of concentrations (Figure 5A, B). Construction of concentration response curves from responses measured at 60 s showed potency of cAMP accumulation for both agonists was the same at each sensor location (Figure 5C, D). Similar conclusions could be drawn by comparing plasma membrane *versus* whole cell cAMP responses (Supplementary Fig. 5A–D).

Detectable accumulation of cAMP at deep intracellular compartments within seconds of agonist application are not easily squared with a model in which agonist-induced GLP-1R redistribution plays a major role in controlling cAMP localisation at the whole organelle level. Rather, our results are more compatible with rapid diffusion of agonist-stimulated cAMP throughout the cell [46].

### 1.6 Differences in endosomal cAMP accumulation with ExD3 *versus* ExF1

**Figure 6:**
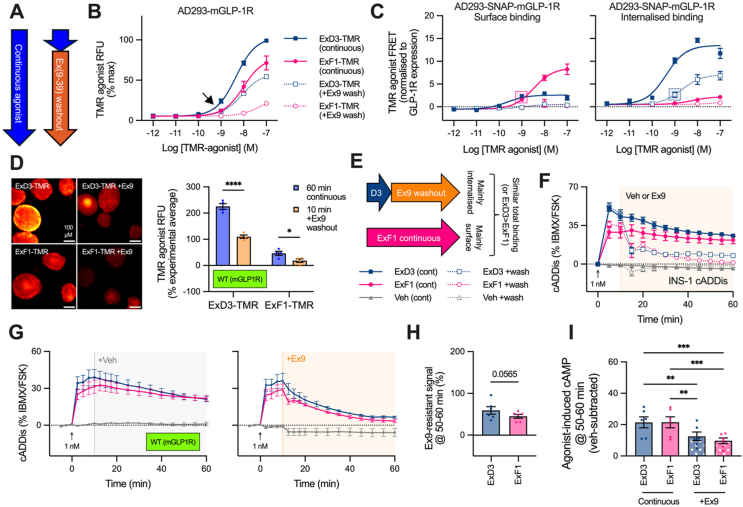
**Assessment of cAMP generation from internalised GLP-1R.** (**A**) Schematic for washout experiment design. (**B**) TMR agonist detection in AD293 cells transiently expressing mGLP1R or hGLP1R with (“+Ex9 wash”) or without (“cont”) 20 μM exendin(9–39) applied 10 min after agonist, *n* = 6. (**C**) Surface and internalised ExD3-TMR and ExF1-TMR binding in AD293 cells expressing SNAP-mGLP-1R, *n* = 6, using same approach as Figure 3C in context of the washout protocol from (A). The shaded squares are intended to highlight how continuously applied ExF1-TMR at 1 nM achieves a similar amount of surface binding to the internalised binding seen with 1 nM ExD3-TMR after washout. (**D**) Representative images and quantification of wild-type mouse islets treated with 1 nM TMR-agonist, with or without exendin(9–39) washout, using the same protocol as in (A). Scale bars = 100 μm. Comparison by two-way matched ANOVA with Sidak's test. (**E**) Graphical summary of how equivalent levels of total ExD3/ExF1 binding can be achieved at different locations. (**F**) Real-time cADDis cAMP responses in INS-1 832/3 cells treated with 1 nM agonist or vehicle, with or without application of 20 μM exendin(9–39) at 10 min, *n* = 5. (**G**) Real-time cADDis cAMP responses in dispersed islets from wild-type C57Bl/6J mice, *n* = 6, treated with 1 nM agonist or vehicle, with or without application of 20 μM exendin(9–39) at 10 min. (**H**) “Exendin(9–39)-resistant” (internalised GLP-1R signalling) sensor responses for the final 10 min from (G), expressed as a percentage of the non-exendin(9–39)-treated response with vehicle-subtraction, are compared by a paired t-test. (**I**) Comparison of vehicle-subtracted responses from the final 10 min from (G) by one-way matched ANOVA with Tukey's test. ∗p < 0.05, ∗∗p < 0.01, ∗∗∗p < 0.001, ∗∗∗∗p < 0.0001 by indicated statistical test. Data represented as mean ± SEM.

Even though data presented above do not support a paradigm in which receptor location is a key determinant of localised cAMP accumulation at the locations tested, this does not preclude the possibility that GLP-1R can generate cAMP from the endosomal compartment. To investigate this, we used a “washout” approach to isolate cAMP responses from internalised receptors, in which an initial period of agonist exposure is followed by application of a high concentration of the cell-impermeable antagonist exendin(9–39) to displace agonist and saturate receptors at the cell surface. As GLP-1R internalisation with ExD3 is almost complete after 10 min (Figure 1E), our protocol involved a 10-minute agonist pulse followed by a 50-minute washout using exendin(9–39) at a supramaximal concentration (20 μM), or maintained agonist exposure throughout the 60-minute stimulation (Figure 6A). This approach was used throughout the experiments presented in Figure 6.

To validate this washout approach, we first measured its effect on TMR-agonist accumulation at 60 min in AD293 cells transfected with untagged mGLP-1R. As expected, exendin(9–39) washout removed the majority of total ExF1-TMR binding, and also substantially reduced that of ExD3-TMR by ∼50% throughout the concentration range (Figure 6B); the latter is presumably because progressive endosomal accumulation of ligand is prevented during the 50-minute washout period. Of note, matched levels of total agonist binding at 60 min were achieved using continuous treatment with 1 nM ExF1-TMR and washout of 1 nM ExD3-TMR (see arrow in Figure 6B); an implementation of the FRET assay from Figure 3 indicated that, under these conditions, ExF1-TMR is mainly at the cell surface and ExD3-TMR is mainly internalised (Figure 6C; relevant data points are highlighted by magenta and blue boxes). Applying the same continuous/washout protocol with 1 nM ExF1-TMR or ExD3-TMR in wild-type mouse islets revealed a similar pattern (Figure 6D), although in this case levels of ExD3-TMR post-washout were higher than those of continuously applied ExF1-TMR, and washout did not completely eliminate all ExF1-TMR binding. The latter could indicate that either 1) a small amount of ExF1-TMR is able to internalise over 10 min before exendin(9–39) is applied, or 2) displacement of surface-bound ExF1-TMR by exendin(9–39) is incomplete under these conditions.

The similar total levels of agonist binding achieved using continuous exposure to 1 nM ExF1 *versus* 1 nM ExD3 plus washout, but at different locations (Figure 6E), provides an opportunity to compare the ability of each ligand/location combination to drive cAMP production. We used cADDis-expressing INS-1 832/3 cells to examine this in the context of endogenous GLP-1R expression (Supplementary Fig. 6A). Here, whilst 1 nM ExF1 produced a smaller cAMP response in the first 10 min than 1 nM ExD3, responses converged later in the incubation with continuous agonist (Figure 6F). In the washout groups, cADDis signal with both ligands declined when exendin(9–39) was applied, but cAMP was better maintained towards the end of the incubation with ExD3 compared to ExF1. Given the almost exclusive endosomal location of ExD3 under these conditions (Figure 6C), this exendin(9–39)-resistant ExD3 cAMP appears to originate from internalised GLP-1R. For ExF1, the exendin(9–39)-resistant portion could arise either from a small amount of internalised ligand or incomplete displacement at the cell surface. However, the fact that continuously applied, predominantly surface-localised ExF1 produced a larger cAMP response than a similar or even larger amount of total, predominantly internalised ExD3 (as per Figure 6E) suggests that endosomal GLP-1R is a less efficient driver than surface GLP-1R for cAMP production, at least when comparing these two agonists. We note that ExF1 is a partial agonist for Gα_s_ [21], which could mean that the difference in signalling potential at each site is even larger than suggested from these data. Supplementary Fig. 6B summarises the effect of washout on cAMP signalling in INS-1 832/3 cells across a range of agonist concentrations.

To extend these observations to primary cells, we used dispersed islets from wild-type C57Bl/6 mice. As with INS-1 832/3 cells, there was clear persistence of the ExD3-induced cAMP signal after washout, and in this case the ExF1 signal was also relatively preserved (Figure 6G). Expressing the vehicle-subtracted cADDis signal from the final 10 min of the incubation after washout relative to that without washout showed a non-significant trend favouring greater exendin(9–39)-resistant cAMP production with ExD3 (Figure 6H). In spite of this trend, it is notable that the apparently much larger amount of ExD3 than ExF1 bound after washout in islets (Figure 6D) did not translate to a commensurate increase in exendin(9–39)-resistant, predominantly endosomal cAMP signalling. Additionally, the washout-resistant ExD3 cAMP response was about half than that achieved by ExF1 at the cell surface when the latter was applied continuously (Figure 6I), even though the former resulted from approximately twice as much total ligand bound when assessed in islets (Figure 6D).

We also used cAMP FRET imaging to measure responses from β- and δ-cells from “CAMPER” mice with cell-specific *Cre*-driven expression of the ^T^Epac^VV^ sensor [47]. Here ExF1 and ExD3 showed similar susceptibility to washout as in β-cells (Supplementary Fig. 6C–E). However, there was a moderate but statistically significant difference between ExF1 and ExD3 exendin(9–39) resistance in δ-cells (Supplementary Fig. 6F–H).

In summary, the washout approach suggests that ExD3 is capable of driving cAMP signalling from endosomes. Careful comparisons of the quantity and location of GLP-1R binding of ExF1 and ExD3 suggest that ExF1 signalling, mainly from the plasma membrane, appears to be more effective at stimulating cAMP production than an equivalent amount of mainly internalised ExD3.

### 1.7 Assessing broader signalling outputs from internalised GLP-1R using a kinome array

To study broader outputs of potential relevance by GLP-1R endosomal signalling, we used the PamChip kinome array to measure activation of a range of kinases in INS-1 832/3 cells in the context of a variant of the washout experiment from Figure 6. The PamChip system measures the ability of agonist-treated cell lysates to phosphorylate of a panel of kinase substrate peptides and has previously been used by us to study GLP-1R signalling in EndoC-βH1 cells [31]. Here we analysed serine–threonine kinase (STK) target peptide phosphorylation by lysates from INS-1 832/3 cells continuously treated by 100 nM agonist over 60 min, or with 10 min agonist plus 50 min exendin(9–39) washout. Because exendin(9–39) might exert signalling effects of its own, we also included an alternative washout protocol in which agonist was removed at 10 min but no antagonist was added. Individual substrate phosphorylation signals and their agonist-induced fold changes compared to vehicle [with or without exendin(9–39)] are shown in Supplementary Fig. 7A and 7B. One biological replicate in the ExF1/exendin(9–39) washout group was considered an outlier on the basis of globally reduced phosphorylation across most substrates, and was excluded from further analysis.

**Figure 7:**
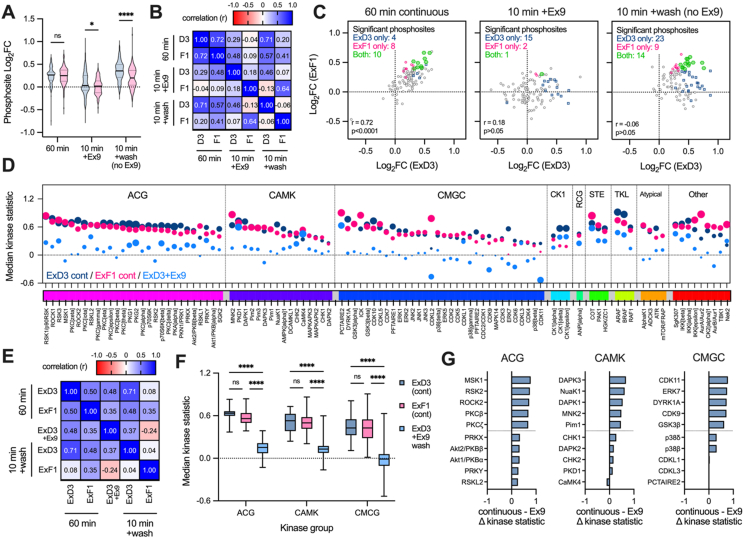
**Assessment of kinase activation from internalised GLP-1R.** (**A**) Violin plots (median, interquartile range, min/max) showing effect of each agonist (100 nM) and washout protocol on substrate phosphorylation derived from *n* = 4 biological replicates. The violin plot captures the log_2_ fold change of each of 110 phosphosites. Comparison by two-way matched ANOVA with Sidak's correction. (**B**) The same data as in (A) but showing the log_2_ fold change for each individual substrate, along with the correlation (Pearson's r) between agonists and whether statistically significant *versus* vehicle for either or both ligands. (**C**) Correlation (Pearson's r) between substrate phosphorylation effects from (A). (**D**) Results of upstream kinase analysis (UKA) to predict activity of 99 kinases using phosphosite data from continuous ExD3, continuous ExF1, and ExD3 post-exendin(9–39)-mediated washout from (A). The direction and magnitude of predicted kinase activity is shown on the y-axis as the “kinase statistic”, and the significance and specificity of the change is represented by the size of the bubbles (“kinase score”). UKA was not performed for ExF1 after exendin(9–39)-mediated washout due to the low number of statistically significant phosphosites. (**E**) Correlation (Pearson's r) between predicted kinase activities from (C). (**F**) Box plots (median, interquartile range, min/max) showing continuous (ExF1, ExD3) and post-exendin(9–39)-mediated washout (ExD3 only) effects of each agonist on the three largest kinase groups (AGC, CAMK and CMCG) by pooling the kinase statistics for each group member, and analysis by two-way matched ANOVA with Sidak's test. (**G**) The top 10 kinases in each group showing the largest (5) and smallest (5) differential between continuous ExD3 and ExD3 post washout, calculated by subtracting the post-washout kinase statistic from the continuous kinase statistic. ∗p < 0.05, ∗∗∗∗p < 0.0001 by indicated statistical test.

60 min continuous stimulation with ExD3 or ExF1 produced a broad (Figure 7A) and well correlated (Figure 7B) overall increase in substrate phosphorylation, with several significant phosphosites shared between agonists (Figure 7C left panel, Supplementary Fig. 7C). Washout using exendin(9–39) reduced net phosphorylation for both ligands, but ExD3 was less affected and several substrates still showed a significant increase in phosphorylation compared to exendin(9–39) alone (Figure 7A–C, Supplementary Fig. 7C). However, noting that data from Figure 6C suggests that continuous treatment with 100 nM ExF1 produces a similar amount of total GLP-1R binding to that achieved by 100 nM ExD3 after exendin(9–39) washout (but with ExF1-TMR mainly at the cell surface and ExD3-TMR mainly internalised), these results again suggest that ExF1 at the plasma membrane produces a larger net effect (p < 0.0001 by paired t-test) on substrate phosphorylation than internalised ExD3 (Figure 7A). Of note, only 4 of the 14 substrates significantly phosphorylated after ExD3 continuous treatment remained significant on exendin(9–39)-induced washout, with additional 11 showing significant phosphorylation activity after washout (Supplementary Fig. 7D), suggesting a distinct signalling profile from the internalised receptor. The alternative non-antagonist mediated washout had a smaller overall effect on suppressing substrate phosphorylation than exendin(9–39), but ExD3 effects were again more resistant to washout (Figure 7A–C, Supplementary Fig. 7C and 7E), indicating relevant GLP-1R signal transduction from endosomal locations.

To aid interpretation of these substrate phosphorylation data, we performed upstream kinase analysis (UKA) to predict the activity of a wide range of kinases using functional class scoring, focussing on the continuous and exendin(9–39)-mediated washout conditions; however, because this approach performs best on datasets with larger numbers of significantly increased phosphosites, we did not include the ExF1 plus exendin(9–39)-induced washout group which contained only 3 significantly phosphorylated substrates. Predicted activities for 99 kinases were determined and are represented in Figure 7D after organisation into groups; equivalent data for the non-antagonist washout groups are shown in Supplementary Fig. 7F. In spite of their opposing signal bias characteristics [20], but in keeping with their similar substrate phosphorylation patterns (Figure 7B), predicted kinase activities for continuous ExF1 and ExD3 treatment were similar and well correlated across all of the kinase groups (Figure 7D, E). We highlight the similar predicted ERK1/2 responses in the CMGC group, in keeping with the data in Figure 4E, which suggests that lack of β-arrestin recruitment with ExF1 does not translate to impaired activity of kinases typically thought to require β-arrestin-mediated activation [4]. After exendin(9–39)-mediated washout, many of the predicted kinases activities induced by ExD3 were lost or reduced across the main kinase groups (Figure 7F), although some were less affected such as PKD1 and CaMK4 in the CaMK group, and PCTAIRE2 and p38 in the CMGC group (Figure 7G). The non-antagonist mediated washout produced a different pattern wherein ExF1 showed disproportionate loss of predicted activity in the ACG and CAMK kinase groups, but moderately greater activity than ExD3 in many kinases in the CMGC group (Supplementary Fig. 7F and 7G).

Overall, these data indicate that the broader kinomic responses induced by 60-minute continuous treatment with ExF1 and ExD3 in INS-1 832/3 cells are rather similar, in keeping with our earlier study comparing these ligands in EndoC-βH1 cells [31]. Moreover, the washout approach again highlighted that the signalling programme attributable to ExD3 signalling from within endosomes was more muted than the GLP-1R dependent response from an equivalent amount of bound ExF1.

### 1.8 Similar effects of ExF1 and ExD3 on islet hormone secretion profiles after agonist washout

**Figure 8:**
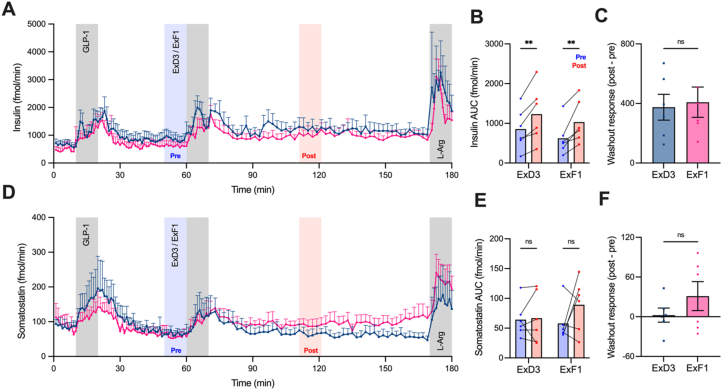
**Hormone release from perfused rat pancreas.** (**A**) Insulin secretion from perfused rat pancreas at 6 mM glucose, *n* = 6. Grey shading indicates perfusion of GLP-1, ExD3, ExF1 (all 1 nM) and l-arginine (10 mM). The blue shading represents the pre-agonist baseline, and the red shading represents 50–60 min after agonist washout. (**B**) Period-normalised AUC calculated from the 10 min before and 50–60 min after perfusion of each agonist, with comparison by two-way repeated measures ANOVA with Sidak's test. (**C**) Comparison by unpaired t-test of the difference between baseline and post-washout insulin secretion with each agonist. (**D**), (**E**) and (**F**) represent the same analyses but for somatostatin. ∗∗p < 0.01 by indicated statistical test. Data represented as mean ± SEM.

To build on these signalling results, we adapted this approach to establish effects on hormone secretion kinetics using the isolated perfused rat pancreas model [48,49]. Here, after an initial exposure to GLP-1(7–36)NH_2_ at 6 mM glucose to confirm the responsiveness of each preparation to GLP-1R agonist stimulation, ExF1 or ExD3 (1 nM) was perfused for 10 min, followed by a prolonged washout period, during which effusate was collected and analysed for insulin and somatostatin. In these experiments washout was achieved by continuous perfusion rather than by addition of exendin(9–39). Examining the traces, it is apparent that insulin secretion after the ExF1/D3 exposure period remains elevated compared to before (Figure 8A). To quantify this effect, hormone secretion before and after 50 min after the agonist exposure period (i.e. matching the signalling time-points from Figure 6, Figure 7) was compared. This showed that both ExF1 and ExD3 were able to significantly and similarly enhance insulin secretion during the washout period (Figure 8B, C). However, whilst both ligands induced an acute increased in somatostatin release, there was no statistically significant effect of either ligand after the washout period (Figure 8D–F).

## 2 Discussion

Here we have investigated the interplay between trafficking and signalling effects of two prototypical biased GLP-1R agonists, ExD3 and ExF1. These ligands have been frequently used to probe the effects of GLP-1R biased agonism due to their similar structure, stability and pharmacokinetics, but divergent signalling properties [20,21,29,30,32,41]. We find that, despite clear and pronounced differences in GLP-1R internalisation with each ligand in cell lines and pancreatic islets, there were no major changes to the subcellular localisation of cAMP signalling when measured at a whole organelle level. Moreover, whilst some of our washout experiments indicate that internalised ExD3/GLP-1R complexes are capable of driving intracellular cAMP signalling, this did not lead to a significant difference in insulin secretion from perfused rat pancreata. Overall these data do not suggest that GLP-1R agonists with a lesser tendency to induce internalisation will fail to engage with cAMP-dependent signalling events deeper into the cell.

GPCR signalling localisation is now a major area of research interest [10]. Inherent to this phenomenon is the assumption that signalling responses are subject to tight spatial control, for example in the form of sharp cAMP concentration gradients close to the site of generation. Indeed, this has been reported when comparing GLP-1-induced cAMP responses at different plasma membrane nanodomains in HEK293 cells with overexpressed GLP-1R [12]. This contrasts somewhat with the current work in which cAMP was rapidly detected at intracellular locations distant from the activated GLP-1R in β-cells expressing GLP-1R endogenously. However, it is possible to rationalise both sets of findings by considering the different conditions tested. cAMP is rapidly diffusible in intact cells [46], but at low, non-stimulated concentrations it remains mainly immobilised at local binding sites [50]. Anton et al. found that very low concentrations of GLP-1 (1 pM) led to cAMP increases within 60 nm of the GLP-1R in HEK293 cells without changes to whole cell or overall plasma membrane cAMP; however, at higher GLP-1 concentrations (1 nM and 100 nM), no such gradient was seen [12]. This suggests that cAMP signalling localisation may be a larger factor in physiological GLP-1 stimulation than with the higher agonist concentrations seen in the pharmacological or therapeutic GLP-1RA setting [22]. β-cells are smaller than many other cell types which could facilitate greater diffusion of cAMP across the cell, and also possess highly specialised cAMP machinery [51], meaning that the impact of GLP-1R localisation could be different outside of a β-cell context. Our studies also used biosensors localised across subcellular organelles, so do not exclude the possibility of steep cAMP gradients existing within smaller nanodomains, for example those closely associated with the GLP-1R itself as in Anton et al. [12], or within receptor and downstream effector-containing multimeric protein complexes (or signalosomes) assembled at inter-organelle membrane contact sites [52,53]. In this instance, and despite widespread cAMP diffusion from other locations, as shown here, effective signal transduction to PKA and its downstream targets might still require localised receptor-containing complex formation. Indeed, we have recently found the latter to be important for driving GLP-1R-mediated β-cell mitochondrial rewiring and lipid handling [54].

Endosomal signalling also provides a potential means to augment total signalling output or duration [8]. Our washout experiment results suggest ExD3 bound to endosomal GLP-1R is indeed a source of cAMP generation, but the size of this response is rather small compared to that produced by an equivalent amount of ExF1 bound mainly at the plasma membrane. We note that ExF1, as well as showing G protein-biased signalling, is also a partial agonist for Gα_s_ when compared to ExD3 [21], which could mean that the above comparison understates the difference in cAMP signalling potential for GLP-1R at the plasma membrane. Nevertheless, whilst ExF1 shows much reduced agonist-induced internalisation, it is not completely absent. Indeed, in islet models a small fraction of ExF1-TMR binding and a somewhat larger fraction of the ExF1-induced cAMP signal was resistant to exendin(9–39)-induced washout. This could be due to incomplete displacement of ExF1 at the cell surface, but it could also indicate efficient cAMP generation by a small amount of internalised ExF1/GLP-1R complexes. If the latter, this would be in keeping with the ability of this ligand to avoid negative regulation e.g. by β-arrestins or rapid transit towards lysosomal degradation [20]. Testing additional ligands could provide a more nuanced comparison if they are better able to leverage endosomal signalling mechanisms than ExD3. Nevertheless, the implication is that, if one wishes to maximise total GLP-1R signalling, there is more to gain from designing ligands with reduced or slower internalisation propensity, unless specific downstream effects emanating from intracellular signalling pools are sought as the desired effect.

In this work we relied on agonist-specific patterns of internalisation to provide selective endosomal or plasma membrane localisation of active GLP-1R. Alternative approaches include pharmacological or genetic inhibition of receptor internalisation, for example the small molecule dynamin inhibitor dyngo4A [55] or overexpression of dominant negative dynamin K44 mutants [56], both of which have been used to probe the importance of GLP-1R internalisation on its signalling responses. For example, co-expression of dynamin-K44E partially inhibited maximal cAMP and ERK1/2 responses to a panel of GLP-1RAs in FlpIn-CHO cells [14], and dyngo4a produced a broad inhibition of GLP-1RA signalling responses in HEK293 cells as measured by phosphoproteomic analysis [16]. An alternative dynamin inhibitor, dynasore, reduced exendin-4-induced cAMP production in BRIN-BD11 clonal β-cells [5]. These effects might give clearer readouts as the inhibition of receptor internalisation would presumably be more complete, but dynamin inhibition may well also result in widespread changes to cellular physiology that extend beyond the GPCR of interest, either by off-target inhibitor binding [57] or as a consequence of inhibition of a multitude of other cellular trafficking processes. A more refined method would involve expressing a mutant GLP-1R unable of being internalised, for example by mutating its intracellular AP2 binding sites.

We focussed in this project on second messenger (and beyond) measures of signalling, i.e. intracellular cAMP, nuclear PKA and ERK1/2, and the broader kinome, meaning that we could assess more proximal signalling events. Indeed, several biosensor-based approaches exist to monitor GPCR-G protein interactions of activation, e.g. TRUPATH [58], ONE-GO [59], EMTA [60] and mini-G proteins [61], with the latter two having been adapted to measure responses at different endosomal compartments [16,62]. However, all these methods require exogenous G protein expression, raising questions as to whether the measured Gα subtype couplings are physiologically meaningful or simply artefacts of overexpression. Moreover, injudicious overexpression of G proteins can disturb GPCR trafficking [63], which is potentially a major issue when using this method to study endosomal signalling. Detection of endogenous G protein activation using either nanobody-37 [9] or BERKY sensors [64] has been reported, with the former used to reveal endosomal Gα_s_ activation by GLP-1R [63]. Nevertheless, measurement of the magnitude and location of second messenger and downstream responses, as in the present work, is essential to put the more proximal events in the signalling chain in context.

Overall, our data suggests that the location of activated GLP-1R has a limited effect on location of cAMP accumulation, at the whole organelle level, in β-cells. We acknowledge some limitations with this work. In addition to the caveats highlighted above, whilst ExF1 and ExD3 are frequently used as tool compounds to provide insights into the mechanisms of action of clinical stage ligands with similar biased signalling and trafficking profiles, they do not capture the full pharmacological attributes that underpin the therapeutic effects of ligands such as tirzepatide, CT-388 etc, including pharmacokinetics and dual targeting of additional receptors. We examined signalling here meanly in pancreatic islet cells; it is possible that intact islets would have behaved differently. Finally, and potentially most importantly, we restricted our experiments to relatively short stimulations, i.e. up to 60 min, aligning with the fast kinetics of GLP-1R endocytosis. However, *in vivo* pharmacology studies show that the apparent therapeutic benefits of G protein-biased GLP-1RAs with reduced internalisation typically may take several hours to emerge [20], meaning that additional approaches will be required to fully understand their mechanism of action.

## CRediT authorship contribution statement

**Shiqian Chen:** Writing – review & editing, Investigation, Formal analysis. **Carolina B. Lobato:** Writing – review & editing, Investigation, Formal analysis. **Carissa Wong:** Writing – review & editing, Investigation, Formal analysis. **Yusman Manchanda:** Writing – review & editing, Investigation, Formal analysis. **Katrina Viloria:** Writing – review & editing, Investigation, Formal analysis. **Iona Davies:** Writing – review & editing, Investigation, Formal analysis. **Daniel B. Andersen:** Writing – review & editing, Investigation, Formal analysis. **Julia Ast:** Writing – review & editing, Resources, Investigation. **Kyle W. Sloop:** Writing – review & editing, Resources. **David J. Hodson:** Writing – review & editing, Resources, Funding acquisition. **Johannes Broichhagen:** Writing – review & editing, Resources, Investigation, Funding acquisition. **Steve Bloom:** Writing – review & editing, Supervision, Funding acquisition. **Jens J. Holst:** Supervision, Funding acquisition. **Tricia Tan:** Writing – review & editing, Supervision, Funding acquisition. **Alejandra Tomas:** Writing – review & editing, Supervision, Resources, Funding acquisition, Conceptualization. **Ben Jones:** Writing – review & editing, Writing – original draft, Supervision, Resources, Investigation, Funding acquisition, Formal analysis, Conceptualization.

## Funding

B.J. acknowledges funding from the Medical Research Council (MR/Y00132X/1 and MR/X021467/1), the Wellcome Trust (301619/Z/23/Z), Diabetes UK, the Eli Lilly LRAP programme, and Metsera Inc. The A.T. lab is funded by an MRC Project Grant (MR/X021467/1) and a Wellcome Trust Discovery Award (301619/Z/23/Z). A.T. also acknowledges funding from Diabetes UK, the Eli Lilly LRAP programme and the Society for Endocrinology. D.J.H. was supported by Diabetes UK (22/0006389) and UKRI ERC Frontier Research Guarantee (EP/X026833/1) Grants. This project has received funding from the European Union#8217; s Horizon Europe Framework Programme (deuterON, grant agreement no. 101042046 to J.B.). This work was supported on behalf of the “Steve Morgan Foundation Type 1 Diabetes Grand Challenge” by Diabetes UK and SMF (grant number 23/0006627 to D.J.H. and J.B.). The Novo Nordisk Foundation Center for Basic Metabolic Research is an independent research center at the University of Copenhagen, partially funded by an unrestricted donation from the Novo Nordisk Foundation (NNF18CC0034900 and NNF23SA0084103). I.D. is funded via a Medical Research Council Doctoral Training Partnership. The Section of Endocrinology and Investigative Medicine at Imperial College London is funded by grants from the MRC, NIHR and is supported by the NIHR Biomedical Research Centre Funding Scheme and the NIHR/Imperial Clinical Research Facility.

## Declaration of competing interest

BJ has received funding from Eli Lilly, Metsera Inc and Sun Pharmaceutical Industries, and acts as a consultant for Metsera Inc. AT has received funding from Eli Lilly and Sun Pharmaceutical Industries. SRB is an employee and shareholder in Metsera, which is developing gut hormone analogues for treatment of metabolic disease. TMMT is a former consultant for and shareholder in Metsera Inc. DJH and J.B. have filed a patent on GLP1R and GIPR chemical probes. D.J.H. and J.B. receive licensing revenue from Celtarys Research for provision of GLP1R/GIPR chemical probes. D.J.H. has filed patents related to type 2 diabetes therapy and GLP1R agonism. KWS is an employee of Eli Lilly & Co.

## Data Availability

Data will be made available on request.

## Methods

Full methodological details are provided in the Supplementary Methods file.
