# Supplementary material for: Spatially diffuse cAMP signalling with oppositely biased GLP-1 receptor agonists in β-cells despite differences in receptor localisation
Source: Mol Metab. 2025 Dec 12;103:102304. doi: 10.1016/j.molmet.2025.102304 (PMC12808597; doi:10.1016/j.molmet.2025.102304)
Supplement: Multimedia component 1 [file mmc1.docx]

**Supplementary Figures**

**Spatially diffuse cAMP signalling with oppositely biased GLP-1 receptor agonists in β-cells despite differences in receptor localisation**

Shiqian Chen^1^, Carolina B Lobato^2,3^, Carissa Wong^1^, Yusman Manchanda^4^, Katrina Viloria^5^, Iona Davies^1^, Daniel B Andersen^2^, Julia Ast^6,7^, Kyle W Sloop^8^, David J Hodson^5^, Johannes Broichhagen^9,10^ Steve Bloom^1^, Jens J Holst^2,11^, Tricia Tan^1^, Alejandra Tomas^4^, Ben Jones^1^.

^1^ Section of Endocrinology, Department of Metabolism, Digestion and Reproduction, Imperial College London, London W12 0NN, UK.

^2^ Department of Biomedical Sciences, Faculty of Health and Medical Sciences, University of Copenhagen, Copenhagen, Denmark.

^3^ Section of Endocrinology, Department of Medicine, Copenhagen University Hospital – Amager and Hvidovre, Hvidovre, Denmark.

^4^ Section of Cell Biology and Functional Genomics, Department of Metabolism, Digestion and Reproduction, Imperial College London, London W12 0NN, UK.

^5^ Oxford Centre for Diabetes, Endocrinology and Metabolism (OCDEM), NIHR Oxford Biomedical Research Centre, Churchill Hospital, Radcliffe Department of Medicine, University of Oxford, Oxford, UK.

^6^ Institute of Metabolism and Systems Research (IMSR), and Centre of Membrane Proteins and Receptors (COMPARE), University of Birmingham, Birmingham, UK.

^7^ Novo Nordisk Research Centre Oxford, Innovation Building, Oxford, UK.

^8^ Diabetes, Obesity and Complications, Lilly Research Laboratories, Eli Lilly and Company, Indianapolis, IN 46285, USA.

^9^ Leibniz-Forschungsinstitut für Molekulare Pharmakologie (FMP), Berlin, Germany.

^10^ Department of Chemical Biology, Max Planck Institute for Medical Research, Heidelberg, Germany.

^11^ Novo Nordisk Foundation Center for Basic Metabolic Research, Faculty of Health and Medical Sciences, University of Copenhagen, Copenhagen, Denmark.

Correspondence: Ben Jones ([ben.jones@imperial.ac.uk](mailto:ben.jones@imperial.ac.uk)) and Alejandra Tomas ([a.tomas-catala@imperial.ac.uk](mailto:a.tomas-catala@imperial.ac.uk)).


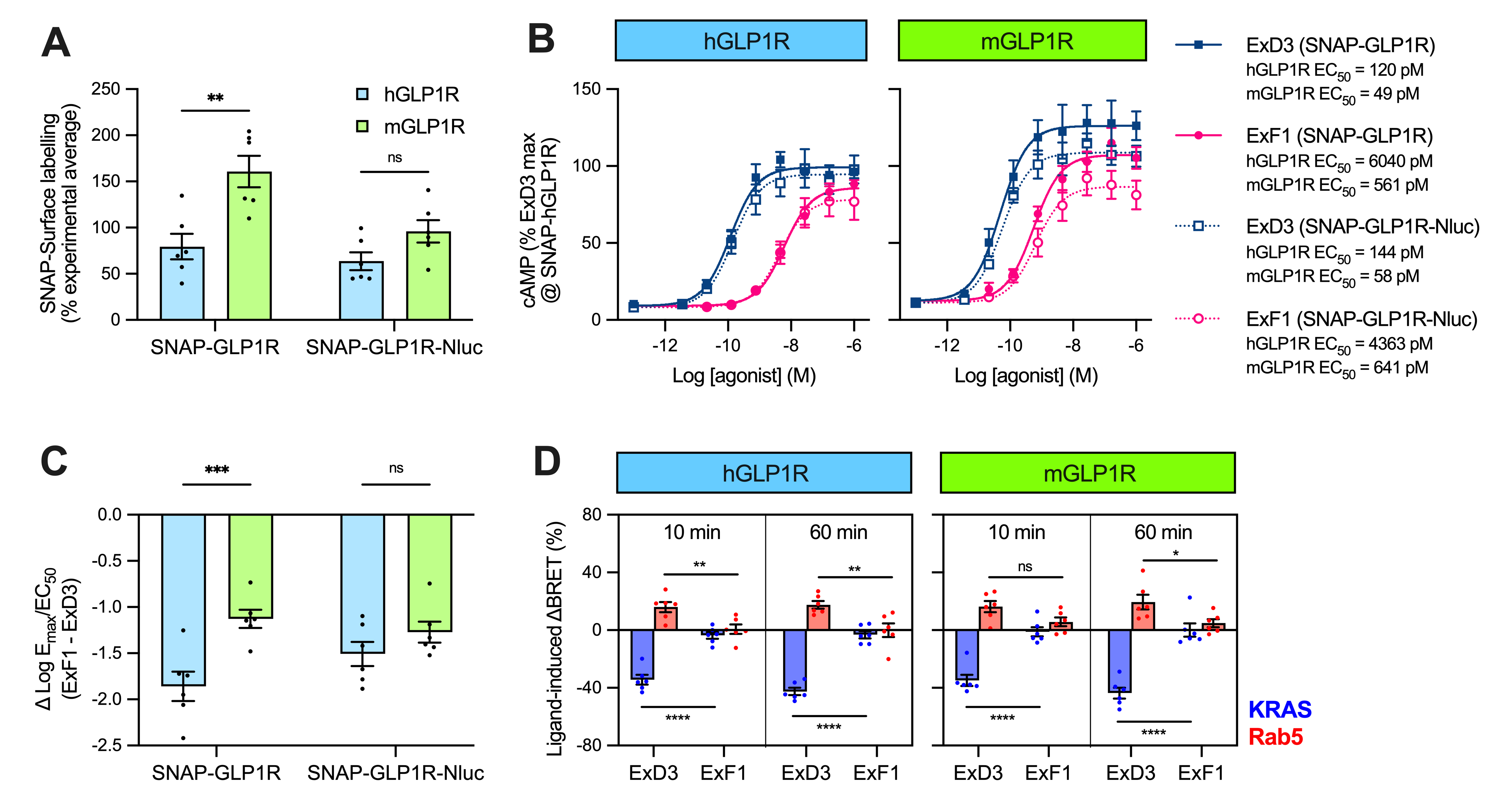


**Supplementary Figure 1.** (**A**) Comparison of SNAP-hGLP-1R, SNAP-mGLP-1R, SNAP-hGLP-1R-Nluc and SNAP-mGLP-1R-Nluc surface expression, measured by SNAP-Surface-AlexaFluor 647 labelling, after transient transfection in AD293 cells, *n*=6, with comparison by two-way matched ANOVA with Sidak’s test. (**B**) cAMP production in transiently transfected AD293 cells, normalised to SNAP-hGLP-1R ExD3 E_max_, *n*=6. (**C**) Comparison of ExF1-specific cAMP signalling at hGLP-1R and mGLP-1R constructs, calculated using ΔLog E_max_/EC_50_ from data in (B), with comparison by two-way matched ANOVA with Sidak’s test. (**D**) Redistribution of GLP-1R in response to 100 nM agonist away from plasma membrane (KRAS) and into early endosomes (Rab5), measured by bystander BRET at two time-points in AD293 cells, *n*=6, with comparison via two-way matched ANOVA with Sidak’s test from vehicle-subtracted AUC. *p<0.05, **p<0.01, ***p<0.0001, ****p<0.0001 by indicated statistical test. Data represented as mean ± SEM.


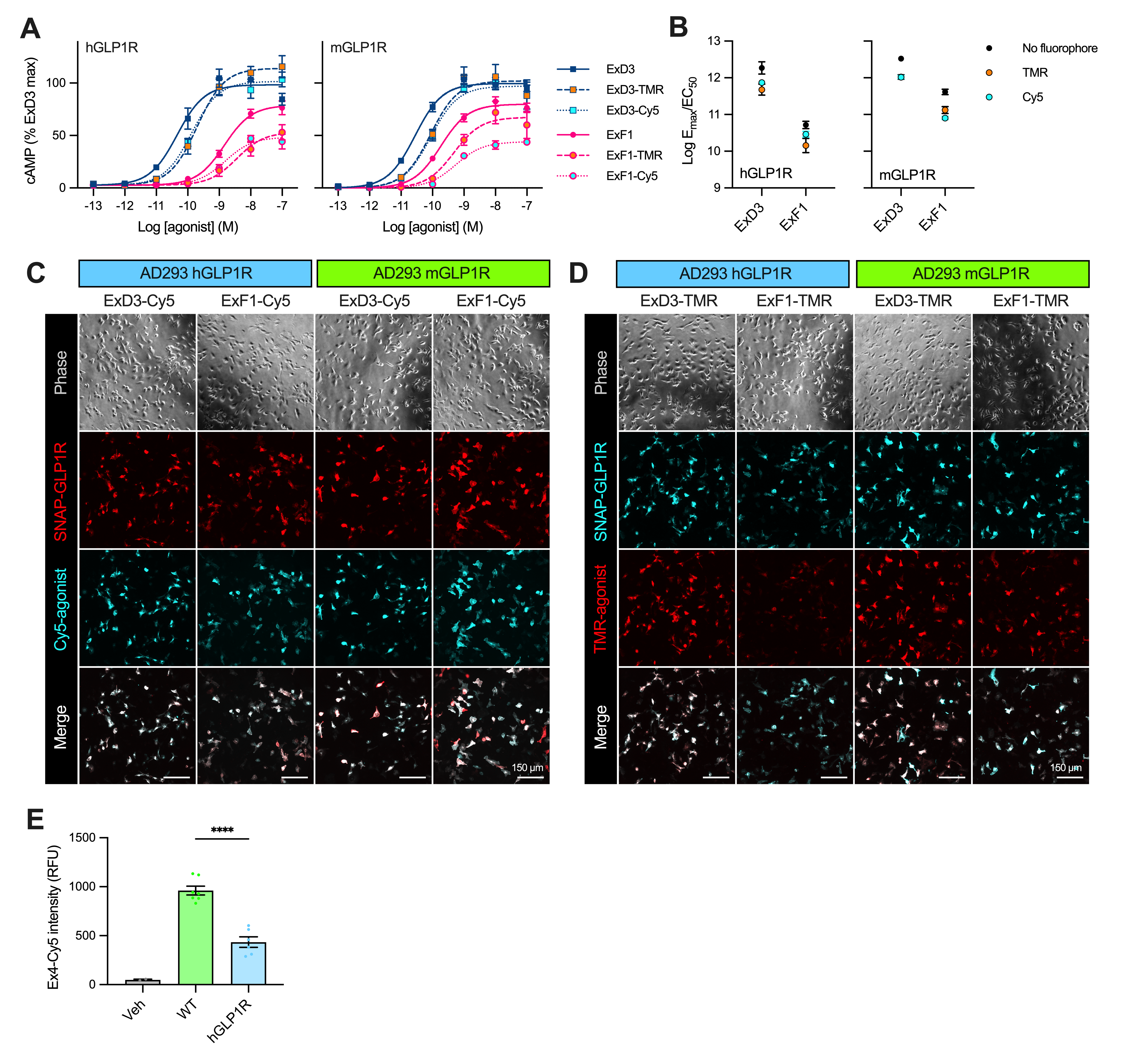


**Supplementary Figure 2.** (**A**) cAMP production in transiently transfected AD293 cells, normalised to SNAP-hGLP-1R ExD3 E_max_, *n*=5. (**B**) Quantification of data from (A) using Log E_max_/EC_50_ method. (**C**) Binding of 3 nM ExF1-Cy5 and ExD3-Cy5 to SNAP-Surface-549-labelled SNAP-GLP-1R and SNAP-GLP-1R in AD293 cells, showing binding only to GLP-1R-positive cells, scale bar = 150 µm. (**D**) As for (C) but using 10 nM ExD3-TMR and 30 nM ExF1-TMR with SNAP-labelling using SNAP-Surface-AlexaFluor 647. (**E**). Comparison of GLP-1R surface expression in islets from wild-type C57Bl/6J (*n*=7) and humanised GLP-1R (*n=*6) mice, assessed by applying 10 nM Ex4-Cy5 for 30 minutes before wide-field imaging, with comparison by unpaired t-test. ****p<0.0001 by indicated statistical test. Data represented as mean ± SEM.


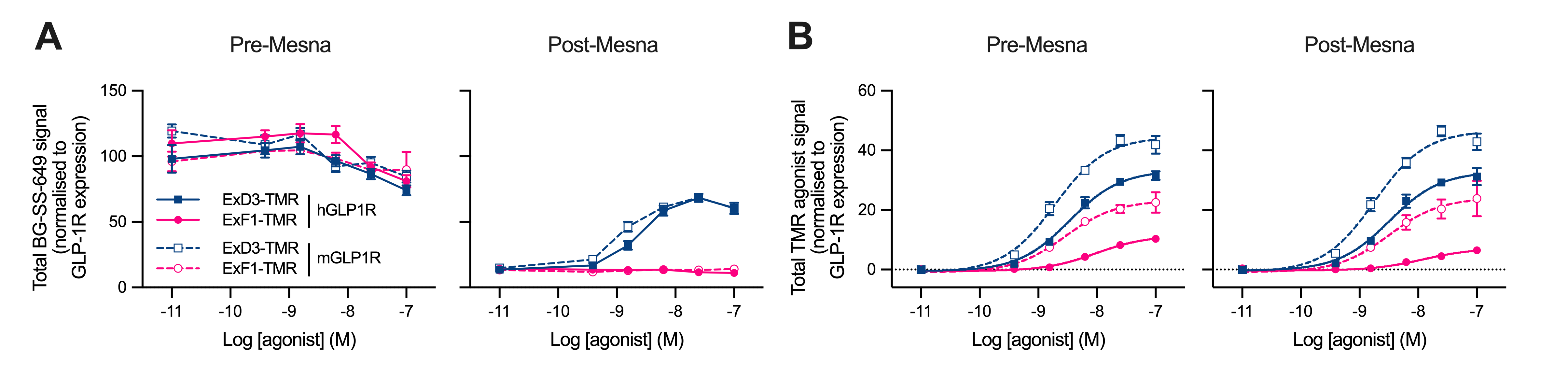


**Supplementary Figure 3.** (**A**) Quantification of receptor labelling with BG-SS-649 before and after Mesna treatment; applies to the experiments shown in Figure 3B-D, *n*=5. (**B**) As for (A) but showing quantification of TMR agonist signal. Data represented as mean ± SEM.


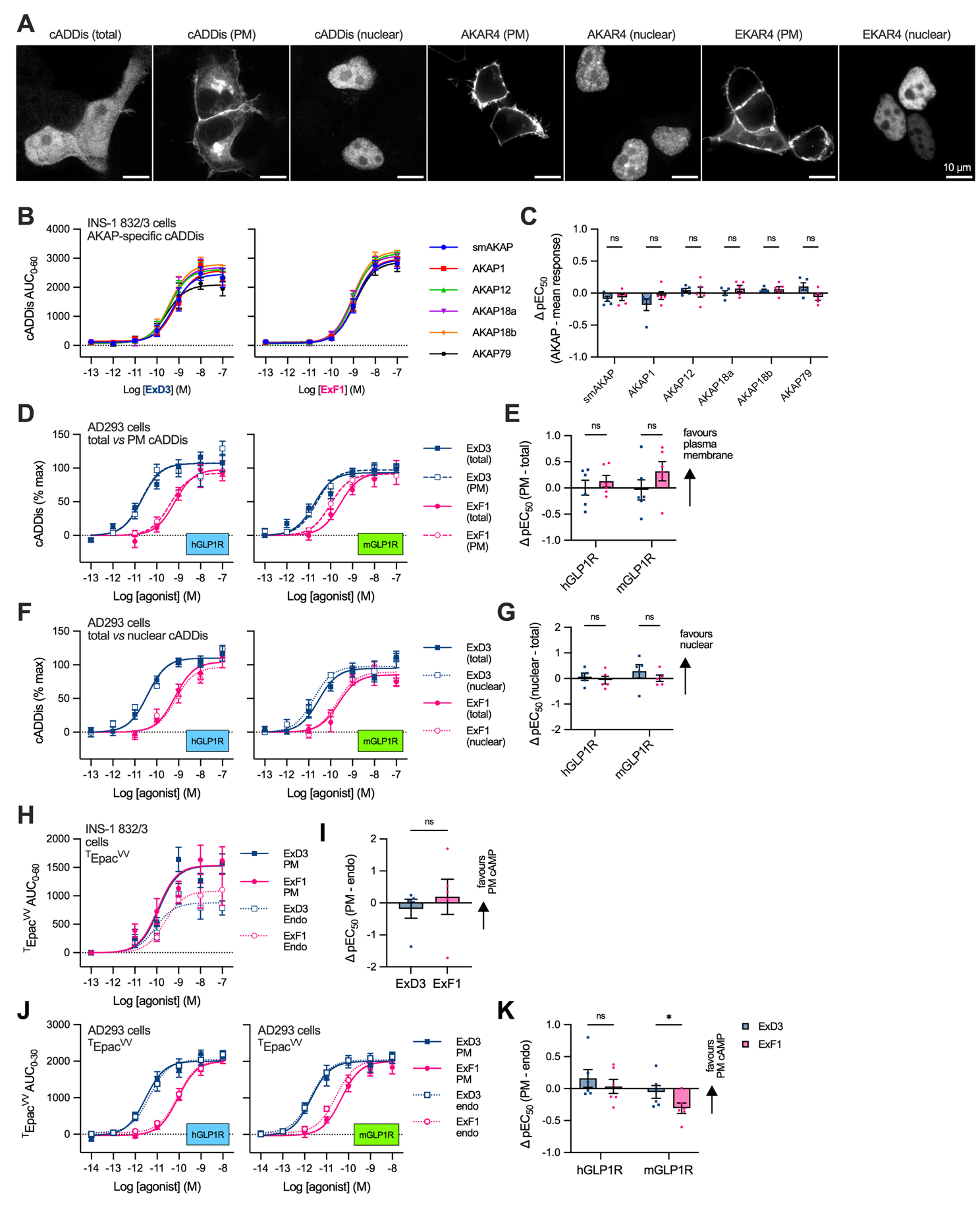


**Supplementary Figure 4. Targeted cAMP biosensor responses in β-cells.** (**A**) High resolution images of cADDis, AKAR4 and EKAR4 sensors used in Fig 4; scale bar = 10 µm. (**B**) AKAP-localised cADDis responses in INS-1 832/3 cells, all tested in parallel in response to 60-min stimulation with ExF1 or ExD3. Concentration responses were established from overall AUC after normalisation to IBMX/FSK control, *n*=5. (**C**) Quantification of AKAP-specific signal localisation by subtracting pEC_50_ for each ligand/AKAP response from (B) from the mean pEC_50_ from all 6 sensors, with comparison by multiple paired t-tests with Holm-Sidak correction. (**D**) Total and plasma membrane (PM) cADDis responses in AD293 cells transiently expressing hGLP-1R or mGLP-1R, 30-min stimulation, *n*=6. (**E**) Quantification of (D) by expressing the pEC_50_ for plasma membrane relative to total cADDis, with comparison by two-way matched ANOVA with Sidak’s test. (**F**) As for (D) but comparing total and nuclear (nuc) cADDis, *n*=5. (**G**) As for (E) but quantifying data from (F). (**H**) ^T^Epac^VV^-PM (plasma membrane) and ^T^Epac^VV^-endo (endosome) responses in INS-1 832/3 cells, 60-min stimulation, *n*=5. (**I**) Quantification of (H) by subtracting endo from PM pEC_50_, with comparison by paired t-test. (**J**) As for (H) but in AD293 cells transiently transfected with hGLP-1R or mGLP-1R, *n*=6. (**K**) Quantification of (J) by subtracting endo from PM pEC_50_, with comparison by two-way matched ANOVA with Sidak’s test. *p<0.05 by indicated statistical test. Data represented as mean ± SEM.


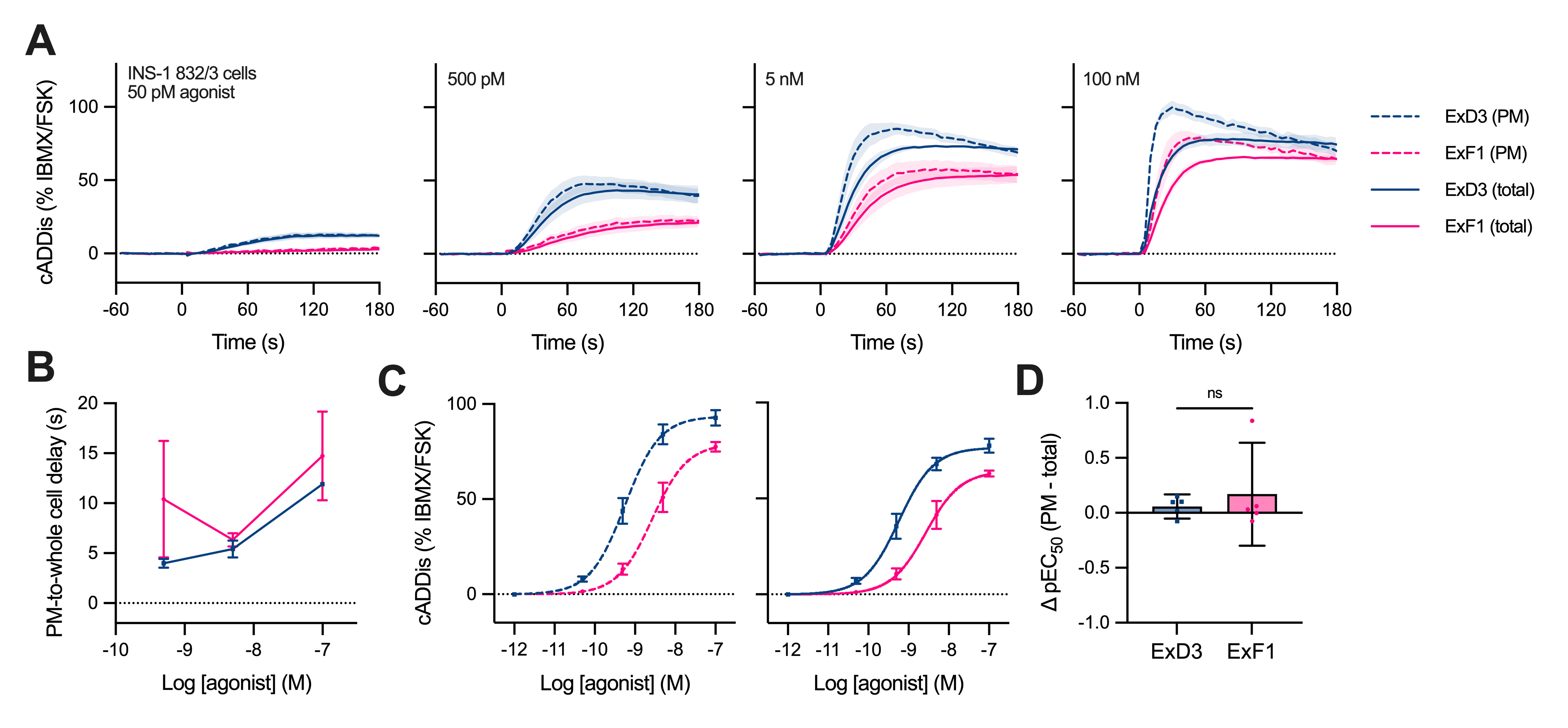


**Supplementary Figure 5. Rapid cAMP kinetics in β-cells.** (**A**) Plasma membrane (PM) and whole cell (total) cAMP kinetic measurements in INS-1 832/3 cells in response to indicated concentration of agonist, *n*=5. (**B**) Delay between plasma membrane and total cAMP estimated by comparing the difference between time-to-half max for each sensor. (**C**) Sensor responses at 60 seconds at several concentrations, with 3-parameter logistic fit. (**D**) Quantification of signal localisation using data from (C), by subtracting pEC_50_ values for PM from whole cell cADDis. 95% confidence intervals are shown, and ExF1 and ExD3 are compared paired t-test. Data represented as mean ± SEM unless indicated.

**
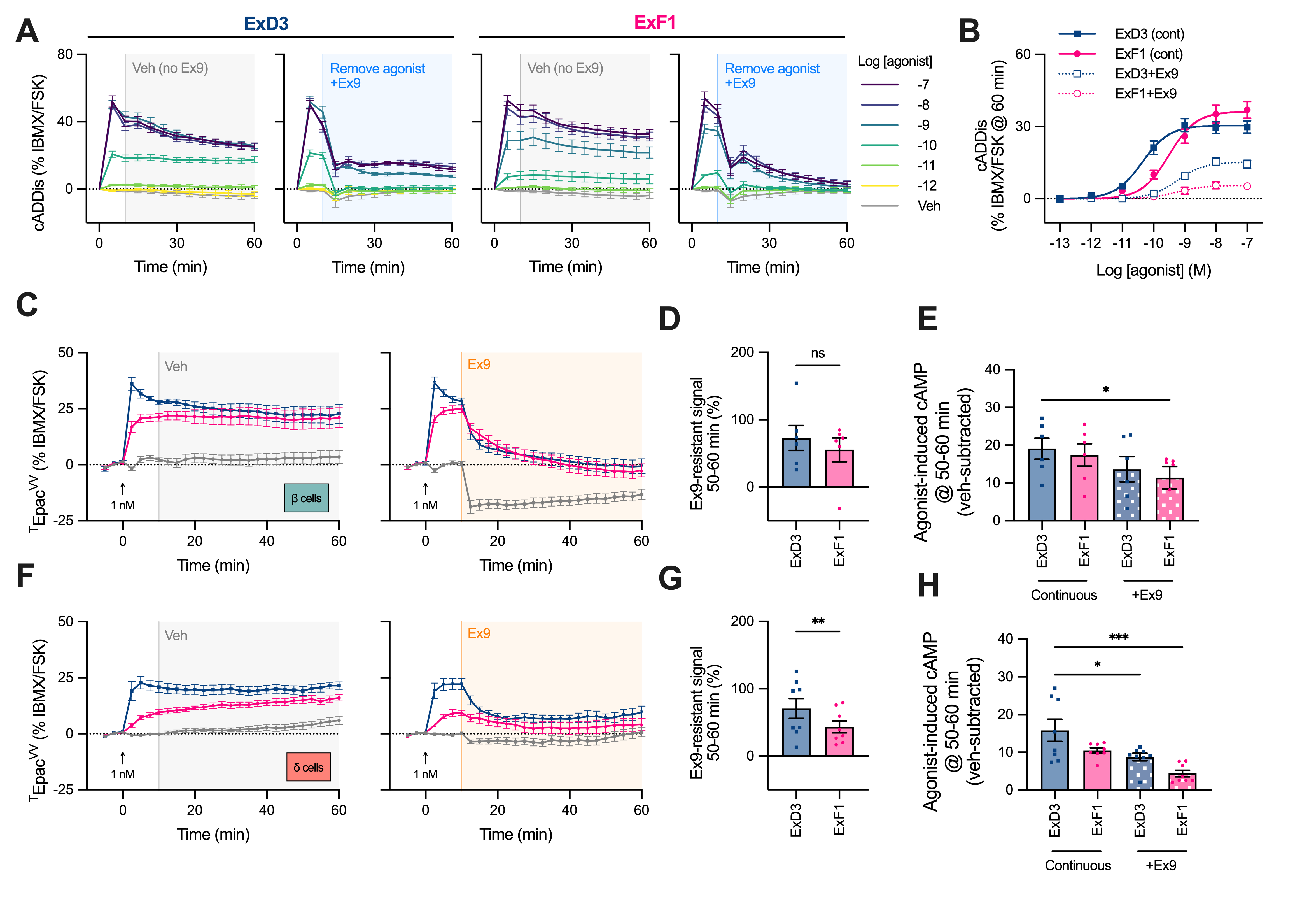
**

**Supplementary Fig 6**. (**A**) Real-time cADDis cAMP responses in INS-1 832/3 cells treated with indicated concentration of agonist or vehicle, with or without application of 20 µM exendin(9-39) at 10 minutes, n=5. (**B**) Concentration response data representing vehicle-subtracted cADDis signal at 60 minutes from the same experiment as Fig 6F. (**C**) cAMP FRET measurements using dispersed islets from Pdx1-Cre^ERT2^/CAMPER mice expressing ^T^Epac^VV^ in β-cells, *n*=6, treated with 1 nM agonist or vehicle, with or without application of 20 µM exendin(9-39) at 10 minutes. (**D**) “Exendin(9-39)-resistant” sensor responses for the final 10 minutes from (C), expressed as a percentage of the non-exendin(9-39)-treated response with vehicle-subtraction, compared by a paired t-test. (**D**) Comparison of vehicle-subtracted responses from the final 10 minutes from (C) by one-way matched ANOVA with Tukey’s test. (**F**), (**G**) and (**H**) show equivalent experiments and analysis as shown in (C), (D) and (E), but using dispersed islets from Sst-Cre/CAMPER mice expressing ^T^Epac^VV^ in δ-cells, *n*=8. *p<0.05, **p<0.01, ***p<0.001 by indicated statistical test. Data represented as mean ± SEM.

**
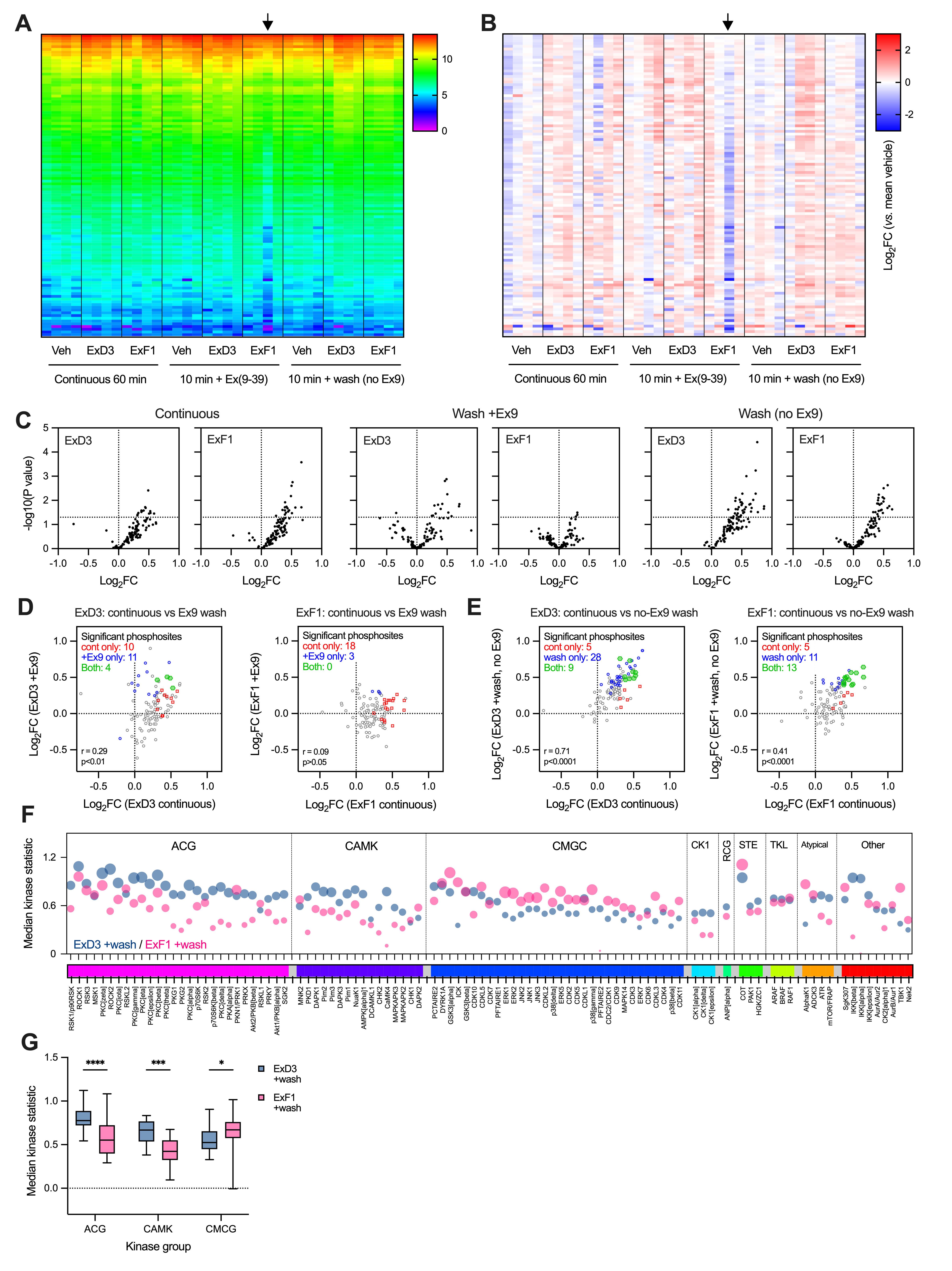
**

**Supplementary Figure 7. Kinome analyses.** (**A**) Heatmap showing individual phosphorylation substrate activity for each of 4 biological replicates. One sample was considered an outlier (arrow). (**B**) The log_2_ fold change of each sample relative to the mean of the 12 vehicle samples. (**C**) Volcano plots showing the log_2_ fold change for each substrate and its p value, relative to the appropriate vehicle group. (**D**) Replotting of data from Fig 7A, showing the log_2_ fold change for each individual substrate with continuous *versus* post-exendin(9-39)-mediated washout, along with the correlation (Pearson’s r) and whether statistically significant *versus* vehicle for either or both ligands. (**E**) As for D but with non-antagonist-mediated washout. (**F**) Results of upstream kinase analysis (UKA) for post-washout (non-Ex9) ExD3 and ExF1 groups; same approach as in Fig 7D. (**F**) Box plots (median, interquartile range, min/max) showing analysis of post-washout (non-Ex9) effects of each agonist on the three largest kinase groups (AGC, CAMK and CMCG) by pooling the kinase statistics for each group member, and analysis by two-way matched ANOVA with Sidak’s test. *p<0.05, ***p<0.01, ****p<0.0001 by indicated statistical test.
