# Supplementary material for: Spatially diffuse cAMP signalling with oppositely biased GLP-1 receptor agonists in β-cells despite differences in receptor localisation
Source: Mol Metab. 2025 Dec 12;103:102304. doi: 10.1016/j.molmet.2025.102304 (PMC12808597; doi:10.1016/j.molmet.2025.102304)
Supplement: Multimedia component 2 [file mmc2.docx]

**Supplementary Methods**

**Spatially diffuse cAMP signalling with oppositely biased GLP-1 receptor agonists in β-cells despite differences in receptor localisation**

Shiqian Chen^1^, Carolina B Lobato^2,3^, Carissa Wong^1^, Yusman Manchanda^4^, Katrina Viloria^5^, Iona Davies^1^, Daniel B Andersen^2^, Julia Ast^6,7^, Kyle W Sloop^8^, David J Hodson^5^, Johannes Broichhagen^9,10^ Steve Bloom^1^, Jens J Holst^2,11^, Tricia Tan^1^, Alejandra Tomas^4^, Ben Jones^1^.

^1^ Section of Endocrinology, Department of Metabolism, Digestion and Reproduction, Imperial College London, London W12 0NN, UK.

^2^ Department of Biomedical Sciences, Faculty of Health and Medical Sciences, University of Copenhagen, Copenhagen, Denmark.

^3^ Section of Endocrinology, Department of Medicine, Copenhagen University Hospital – Amager and Hvidovre, Hvidovre, Denmark.

^4^ Section of Cell Biology and Functional Genomics, Department of Metabolism, Digestion and Reproduction, Imperial College London, London W12 0NN, UK.

^5^ Oxford Centre for Diabetes, Endocrinology and Metabolism (OCDEM), NIHR Oxford Biomedical Research Centre, Churchill Hospital, Radcliffe Department of Medicine, University of Oxford, Oxford, UK.

^6^ Institute of Metabolism and Systems Research (IMSR), and Centre of Membrane Proteins and Receptors (COMPARE), University of Birmingham, Birmingham, UK.

^7^ Novo Nordisk Research Centre Oxford, Innovation Building, Oxford, UK.

^8^ Diabetes, Obesity and Complications, Lilly Research Laboratories, Eli Lilly and Company, Indianapolis, IN 46285, USA.

^9^ Leibniz-Forschungsinstitut für Molekulare Pharmakologie (FMP), Berlin, Germany.

^10^ Department of Chemical Biology, Max Planck Institute for Medical Research, Heidelberg, Germany.

^11^ Novo Nordisk Foundation Center for Basic Metabolic Research, Faculty of Health and Medical Sciences, University of Copenhagen, Copenhagen, Denmark.

Correspondence: Ben Jones ([ben.jones@imperial.ac.uk](mailto:ben.jones@imperial.ac.uk)) and Alejandra Tomas ([a.tomas-catala@imperial.ac.uk](mailto:a.tomas-catala@imperial.ac.uk)).

# Methods

## Peptides and general chemistry

ExF1, ExD3, ExF1-TMR and ExD3-TMR were purchased WuXi AppTec, China. Ex4-S39C, ExF1-S39C and ExD3-S39C, both from Wuxi Apptec, were used to prepare Ex4-Cy5, ExF1-Cy5 and ExD3-Cy5 by cysteine-maleimide conjugation [1].

### General chemistry

RP-HPLC was performed on a Waters e2695 system equipped with a 2998 PDA detector for product collection (at 650 nm) on a Supelco Ascentis® C18 HPLC Column (5 μm, 250 × 10 mm). Buffer A: 0.1% TFA in H_2_O Buffer B: acetonitrile. The typical gradient was from 10% B for 5 min 🡪 gradient to 90% B over 45 min 🡪 90% B for 5 min 🡪 gradient to 99% B over 5 min with 4 mL/min. High resolution mass spectrometry was performed using a Bruker maXis II ETD hyphenated with a Shimadzu Nexera system. The instruments were controlled *via* Brukers otofControl 4.1 and Hystar 4.1 SR2 (4.1.31.1) software. The acquisition rate was set to 3 Hz and the following source parameters were used for positive mode electrospray ionization: End plate offset = 500 V; capillary voltage = 3800 V; nebulizer gas pressure = 45 psi; dry gas flow = 10 L/min; dry temperature = 250 °C. Transfer, quadrupole and collision cell settings are mass range dependent and were fine-adjusted with consideration of the respective analyte’s molecular weight. For internal calibration sodium format clusters were used. Samples were desalted *via* fast liquid chromatography. A Supelco Titan^TM^ C18 UHPLC Column, 1.9 µm, 80 Å pore size, 20 × 2.1 mm and a 2 min gradient from 10 to 98% aqueous MeCN with 0.1% FA (H_2_O: Carl Roth GmbH + Co. KG ROTISOLV® Ultra LC-MS; MeCN: Merck KGaA LiChrosolv® Acetonitrile hypergrade for LC-MS; FA - Merck KGaA LiChropur® Formic acid 98%-100% for LC-MS) was used for separation. Sample dilution in 10% aqueous MeCN (hyper grade) and injection volumes were chosen dependent of the analyte’s ionization efficiency. Hence, on-column loadings resulted between 0.25–5.0 ng. Automated internal re-calibration and data analysis of the recorded spectra were performed with Bruker’s DataAnalysis 4.4 SR1 software.

### Synthesis of Ex4(1-39)_S39C_Cy5

To a solution of Ex4(1-39)_S39C (Wuxi Apptec, 900 μg, 214 nmol, 1.0 eq.) in PBS (500 uL) was added Cy5-Mal (Lumiprobe #43020, 321 nmol, 1.5 eq.). The solution was incubated on a shaker (500 rpm) at 37 °C over night before being subjected to RP-HPLC purification. The purified fractions were combined, the concentration was determined *via* the absorption of the Cy5 fluorophore at 647 nm (ε = 250,000 M ^-1^ cm^-1^ in PBS) and aliquots of 5 nmol each were prepared and lyophilized to yield **Ex4(1-39)_S39C_Cy5** as a deep blue powder.

**HRMS** (ESI): calc. for C_222_H_332_N_54_O_62_S_2_ [M+5H]^6+^: 802.0658, found: 802.0659.

### Synthesis of Ex4(1-39)_H1F_S39C_Cy5

To a solution of Ex4(1-39)_H1F_S39C (Wuxi Apptec, 1.3 mg, 298 nmol, 1.0 eq.) in PBS (500 uL) was added Cy5-Mal (Lumiprobe #43020, 448 nmol, 1.5 eq.). The solution was incubated on a shaker (500 rpm) at 37 °C over night before being subjected to RP-HPLC purification. The purified fractions were combined, the concentration was determined *via* the absorption of the Cy5 fluorophore at 647 nm (ε = 250,000 M ^-1^ cm^-1^ in PBS) and aliquots of 5 nmol each were prepared and lyophilized to yield **Ex4(1-39)_H1F_S39C_Cy5** as a deep blue powder.

**HRMS** (ESI): calc. for C_221_H_330_N_54_O_62_S_2_ [M+5H]^6+^: 799.7299, found: 799.7293.

### Synthesis of Ex4(1-39)_E3D_S39C_Cy5

To a solution of Ex4(1-39)_E3D_S39C (Wuxi Apptec, 2900 μg, 214 nmol, 1.0 eq.) in PBS (500 uL) was added Cy5-Mal (Lumiprobe #43020, 321 nmol, 1.5 eq.). The solution was incubated on a shaker (500 rpm) at 37 °C over night before being subjected to RP-HPLC purification. The purified fractions were combined, the concentration was determined *via* the absorption of the Cy5 fluorophore at 647 nm (ε = 250,000 M ^-1^ cm^-1^ in PBS) and aliquots of 5 nmol each were prepared and lyophilized to yield **Ex4(1-39)_E3D_S39C_Cy5** as a deep blue powder.

**HRMS** (ESI): calc. for C_225_H_333_N_52_O_62_S_2_ [M+4H]^5+^: 924.2795, found: 924.2783.

## Plasmids

pcDNA5/FRT (Thermo Fisher, UK) encoding full length, wild-type human or mouse GLP-1R, and pcDNA5/FRT/TO (Thermo Fisher, UK) encoding human or mouse GLP-1R with an N-terminal SNAP tag with custom signal peptide, and the same but featuring C-terminal nanoluciferase, were generated by Genewiz, UK. KRAS-venus and Rab5-venus were kindly provided by Nevin Lambert (Augusta University, USA). pcDNA3-AKAR4-KRAS, pcDNA3-AKAR4-NLS, pcDNA3-pmEKAR4 and pcDNA3-nuclear-EKAR4 (Addgene plasmids #61621, #138217, #174440 and #174439, respectively) were a gift from Jin Zhang [2–4]. The modification of ^T^Epac^VV^ [5] for localisation to plasma membrane or endosomes was performed *in house* and has been described previously [6].

## Cell lines and cell culture

Adherent HEK293 cells (AD293) were cultured in DMEM supplemented with 10% foetal bovine serum (FBS) and 1% penicillin/streptomycin. INS-1 832/3 cells [7], a gift from Prof Newgard (Duke University, USA), and INS-1 832/3 cells with deletion of the endogenous GLP-1R by CRISPR/Cas9 [8], a gift from Jacqueline Naylor (AstraZeneca, UK), and were cultured in RPMI with 10% FBS and 1% penicillin/streptomycin.

## Animals

All mouse procedures were approved by the UK Home Office under the UK Animals (Scientific Procedures) Act 1986. The generation of GLP1R^SNAP/SNAP^ mice [9] and humanised GLP-1R mice [10] has been described previously. Mice expressing the cAMP FRET sensor ^T^Epac^VV^ in specific tissues were generated by breeding CAMPER mice [11] (C57BL/6-*Gt(ROSA)26Sor^tm1(CAG-ECFP*/Rapgef3/Venus*)Kama^*/J; Jax strain #032205) with appropriate *Cre* strains including *Pdx1-CreERT2* [12] (*Tg(Pdx1-cre/Esr1*)#Dam/J*; Jax strain #024968) and *Sst-Cre* [13] (*Sst^tm2.1(cre)Zjh^/J*; Jax strain #013044). Wild-type C57BL/6J mice were purchased from Charles River, UK. Mice were group housed in a condition-controlled room at 21-23°C with 12h:12h light to dark cycles (lights on at 07:00). All animals had *ad libitum* access to water and RM1(E) feed (Special Diet Services, UK).

## Pancreatic islet isolation

Pancreas were inflated through the common bile duct with 1 mg/ml collagenase (Universal Biologicals Ltd, UK) in RPMI-1640 medium. The pancreas was then dissected and digested at 37°C in a water bath for 10 minutes. Digestion was quenched with cold RPMI-1640 medium supplemented with 10% FBS and 1% penicillin/streptomycin. Islets were then purified using a Histopaque gradient (Histopaque-1119 and -1083, Sigma-Aldrich, UK). Islets were left to recovered overnight at 37°C in 5% CO_2_ in RPMI-1640 containing 10% FBS and 1% penicillin/streptomycin prior to all the experiments. For experiments using dispersed islets, dispersal was performed by triturating islets in 0.25% EDTA/trypsin solution for 3 minutes at 37°C.

## Measurement of cell surface GLP-1R expression in AD293 cells

AD293 cells were reverse transfected with SNAP-tagged hGLP-1R or mGLP-1R for 24 hours using Lipofectamine 2000 (Thermo Fisher, UK) in black-walled, clear-bottomed plates (Greiner, UK) coated with 0.01% poly-D-lysine. Cells were labelled for 10 minutes using SNAP-Surface-AlexaFluor-647 (500 nM) in complete media at 37°C. After washing twice with PBS, cells were washed three times with PBS, followed by imaging using an automated Nikon Ti2-E-based microscope (Cairn Research, UK) using with a LED lightsource (CoolLED, UK) using a 20X air objective Images were analysed using Fiji v1.54f (NIH, USA). Several epifluorescence and transmitted light phase contrast fields-of-view (FOVs) were acquired from each well using the automated stage to cycle through position on the microplate. Cell-associated fluorescence intensity, indicating surface GLP-1R expression, was analysed by applying a flat-field correction and segmentation of cell-containing regions using Phantast [14].

## Biochemical cAMP assays

AD293 cells were reverse transfected with SNAP-tagged hGLP-1R or mGLP-1R for 24 hours using Lipofectamine 2000 in 12-well plates. Cells were detached using EDTA, pelleted, and resuspended in serum-free DMEM with 0.1% BSA before dispensing into 384-well plates containing agonists for 30-minutes. cAMP detection reagents (Cisbio, France) were added prior to reading by HTRF using an Spectramax i3x plate reader.

## Confocal imaging of SNAP-tagged GLP-1R distribution in AD293 cells

AD293 cells were reverse transfected with SNAP-tagged hGLP-1R or mGLP-1R for 24 hours using Lipofectamine 2000 in black-walled, clear-bottomed plates (Ibidi, Germany) coated with 0.01% poly-D-lysine. Cells were labelled for 10 minutes using SNAP-Surface-488 (500 nM) in complete media at 37°C. After washing twice with PBS, agonists (100 nM) were added in KRBH buffer containing 0.1% BSA and 6 mM glucose for 30 minutes at 37°C. For the final 10 minutes, Hoechst dye (1:2000; Thermo Fisher, UK) was added. At the end of the incubation, cells were washed once with PBS, followed by fixation for 10 minutes with 4% paraformaldehyde (PFA). After further washes, cells were imaged using the same microscope as in Section 4.6 but using a laser light source (LDI-7, 89North, USA), spinning disc (X-Light V2, CrestOptics, Italy), and 100X 1.45 NA oil immersion objective. Images were analysed using Fiji v1.54f (NIH, USA).

## Reversible labelling assay to quantify GLP-1R internalisation

AD293 cells were reverse transfected with SNAP-tagged hGLP-1R or mGLP-1R for 24 hours using Lipofectamine 2000 in black-walled, clear-bottomed plates (Greiner, UK) coated with 0.01% poly-D-lysine. Cells were labelled with the cleavable SNAP-tag labelling probe BG-SS-649 (500 nM, a gift from New England Biolabs) for 10 min at 37°C, washed once with PBS, and treated with agonist in serum-free medium containing 0.1% BSA for 30 min at 37°C to induce GLP-1R internalisation. Cells were then washed twice with PBS and placed in Tris/NaCl/EDTA (TNE) buffer, pH 8.6 for imaging. Cells were then imaged before and after addition of the cell-impermeable reducing agent sodium 2-mercaptoethanesulfonate (Mesna; 100 mM) using the same microscope as in Section 4.6, using LED and brightfield illumination via a 20X phase contrast air objective. Cell-associated fluorescence intensity was quantified using the same procedure as in Section 4.6; percentage GLP-1R internalisation was quantified from the pre- and post-Mesna fluorescence, representing total and internalised GLP-1R, respectively, and analysed from 3-parameter logistic fitting.

## Endosomal GLP-1R translocation assay

AD293 cells were reverse transfected with SNAP-tagged hGLP-1R or mGLP-1R for 24 hours using Lipofectamine 2000 in black-walled, clear-bottomed plates (Greiner, UK) coated with 0.01% poly-D-lysine. Cells were labelled with 500 nM SNAP-Surface-549 for 10 min at 37°C, with the labelling media then washed off and replaced with KRBH including 6 mM glucose and 0.1% BSA. Time-lapse imaging was then performed using the same automated microscope platform as in Section 4.6 using LED illumination and a 40X, 0.9 NA air objective, with several fluorescence images per well acquired at set intervals before and after agonist addition. After flat-field correction, endosomal puncta were quantified by Laplacian filtering using FeatureJ (https://imagescience.org/meijering/software/featurej/), thresholding and particle counting in Fiji. Change over time was determined from the fold increase in number of endosomal puncta compared to baseline.

## NanoBRET assay for detection of GLP-1R at the plasma membrane and early endosomes

AD293 cells were reverse transfected with N-terminally SNAP-tagged and C-terminally nanoluciferase tagged hGLP1R or mGLP1R plasmids, plus venus-tagged KRAS or Rab5 markers (1:3 ratio) using Lipofectamine 2000 for 24 hours. For end-point assays, cells were then detached, pelleted, and resuspended in KRBH buffer containing 6 mM glucose and 0.1% BSA, before transfer to opaque white 96-well plates (Greiner) containing agonist or vehicle and, after the indicated stimulation period at 37°C, furimazine (1:100; Promega, UK) was added to each well and BRET signal was recorded using a Glomax plate reader (Promega, UK) at 37°C, with a 460 nm bandpass and 525 nm longpass filter for donor and acceptor, respectively. For kinetic assays, cells were resuspended in KRBH buffer containing 6 mM glucose and 0.1% BSA supplemented with furimazine (1:100) and transferred to opaque white 96-well plates before transfer to the plate reader; BRET signal was then recorded at regular intervals before and after agonist addition. Specific BRET ratio was calculated from the acceptor:donor emission ratio, with subtraction of the ratio obtained from luciferase alone, and expressed as a percentage change relative to vehicle treatment (end-point assays) or to baseline (kinetic assays).

## Confocal imaging of SNAP-tagged GLP-1R distribution in intact pancreatic islets

Generation of adenoviruses expressing SNAP-tagged human GLP-1R was outsourced at VectorBuilder. An adenoviral construct (AV-SNAP-hGLP1R) was custom made from pSNAP-GLP-1R (Revvity) and islets extracted from wild-type mice were infected overnight with an MOI of 1 in full media prior to SNAP-tag labelling with SNAP-Surface-549 for 8 minutes, stimulation with the indicated GLP-1RA and subsequent imaging in a Leica Stellaris 8 inverted confocal microscope with a 20x0.75 air objective from the Facility for Imaging by Light Microscopy (FILM) at Imperial College London. GLP1R^SNAP/SNAP^ islets were incubated with 1.5 µM BG-Sulfo646 [15] for 30 minutes at 37°C RPMI growth medium. After washing 2x with PBS, islets were incubated in fresh media supplemented with ExD3 (100 nM), ExF1 (100 nM), or vehicle at 37°C for another 30 minutes. Imaging was performed using a Zeiss NLO880 confocal microscope with W Plan-Apochromat 20x/1.0 DIC objective. Excitation / detection wavelengths of 633 nm / 639-705 nm were used.

## Fluorescent agonist binding quantification

AD293 cells were reverse transfected with untagged (or, for some experiments, SNAP-tagged) hGLP-1R or mGLP-1R for 24 hours using Lipofectamine 2000 in black-walled, clear-bottomed plates (Greiner, UK) coated with 0.01% poly-D-lysine. Wild-type INS-1 832/3 or INS-1 Glp1r ^-/-^ cells were seeded similarly but without transfection. After removing culture media, cells were treated with a range of Cy5- or TMR-agonist concentrations for 30 minutes; for SNAP-GLP-1R cells agonist treatment was preceded by labelling with SNAP-Surface probes as in Section 4.6. Cells were then briefly washed twice with PBS before fixation with 4% PFA for 10 minutes, followed by two further washes. Cells were then imaged using the same microscope as in Section 4.6 using LED and brightfield illumination via a 20X phase contrast air objective. Fluorescent agonist binding was quantified as in Section X. For INS-1 832/3 cells, endogenous GLP-1R expression is much lower and non-specific signal begins to contribute at high agonist concentrations; therefore, signal from concurrently treated INS-1 Glp1r ^-/-^ cells was subtracted to obtain specific binding. Binding affinity was calculated using a one site specific binding model.

## Fluorescent agonist high resolution imaging

The experiments were performed similarly to as in Section 4.8, except untagged GLP-1R was used and no SNAP-labelling was performed. Fixed cells were imaged using the same microscope as in Section 4.6 using in laser spinning disc configuration and 100X 1.45 NA oil immersion objective. Images were analysed using Fiji v1.54f.

## Confocal imaging of fluorescent agonist distribution in intact pancreatic islets

Intact pancreatic islets from wild-type C57Bl/6J or humanised GLP-1R mice were treated with fluorescent Cy5- or TMR-conjugated agonists for 30 min at 37°C KRBH buffer containing 6 mM glucose and 0.1% BSA, washed, and then imaged with a Zeiss LSM-780 inverted confocal laser-scanning microscope in a ×63/1.4 numerical aperture oil-immersion objective.

## FRET imaging of agonist/receptor interaction via acceptor photobleaching

The experiment was performed as in Section 4.8, except that TMR agonists were used, and the SNAP-Surface-AlexaFluor 647 probe was present during the agonist exposure period rather than washed off first. After fixation, cells were imaged using the same microscope as in Section 4.6 using in laser spinning disc configuration and 40X 1.3 NA oil immersion objective. Confocal images were taken before and after a photobleaching of the SNAP-Surface probe by a 30-second exposure to the 640 nm laser at full power in epifluorescence mode. FRET signal was determined by subtracting or dividing the before-bleaching and after-bleaching images using Fiji v1.54f.

## Ratiometric FRET imaging of agonist/receptor interaction

AD293 cells were reverse transfected with SNAP-tagged hGLP-1R or mGLP-1R for 24 hours using Lipofectamine 2000 in black-walled, clear-bottomed plates (Greiner, UK) coated with 0.01% poly-D-lysine. After labelling with BG-SS-649 for 10 minutes at 37°C, TMR agonist or vehicle was supplemented to the target concentration. For washout experiments, some wells had 20 µM exendin(9-39) applied, with or without agonist removal as described. BG-SS-649 was present throughout the incubations. Cells were then briefly washed twice in cold PBS before being placed in TNE buffer, pH 8.6 for imaging. Ratiometric FRET imaging was performed before and after addition of 100 mM Mesna using the same microscope as in Section 4.6, with an image splitter (Optosplit III, Cairn Research Ltd, UK) to collect TRITC and Cy5 emission spectra. Image analysis was performed using Fiji v1.54f. After flat-field correction and background subtraction, the direct BG-SS-649 image was used to correct the TMR-agonist FRET signal for bleed-through. Phase-contrast images were used to segment cell-containing regions, and direct TMR signal, direct Cy5 signal, and mean TMR FRET signal were calculated. The pre-Mesna TMR FRET signal represents total GLP-1R-bound agonist, post-Mesna FRET signal represents internalised GLP-1R-bound agonist, and the difference between the two represents surface GLP-1R-bound agonist.

## cAMP imaging using cADDis sensors

cADDis sensors in a BacMam vector (Montana Molecular, USA) were transduced according to the manufacturer’s instructions into wild-type INS-1 832/3 cells, AD293 cells, or into dispersed mouse pancreatic islet cells. Sensors included were whole-cell (total) cADDis (U0250G), plasma membrane targeted cADDis (U0241R), nuclear-targeted cADDis (D0221G), and AKAP-targeted cADDis (U0261G, U0262G, U0263G, U0264G, U0266G, U0267G). Cells were seeded into black-walled, clear bottomed 96- or 384-well plates (Greiner) coated with 0.01% poly-D-lysine at the time of transfection/transduction. 24 hours later, media was removed and replaced with KRBH buffer containing 6 mM glucose and 0.1% BSA for 20 min before the assay. Imaging was performed using the same microscope as in Section 4.6 using 10X or 20X air objectives and LED illumination for green and red channels. The automated stage was used to sequentially image several FOVs per well at regular intervals before or after agonist addition; typically, many hundreds of cells were captured from each well. For “washout” experiments, exendin(9-39) was added to some wells to a target concentration of 20 µM; in some cases the agonist was removed prior to exendin(9-39) addition, as described in the results. At the end of the assay 500 µM 3-isobutyl-1-methylxanthine (IBMX; Sigma, UK) and 50 µM forskolin (FSK) was added to generate a maximum sensor response. Individual cell responses were analysed using custom scripts in Fiji v1.54f, with debris or dead cells excluded based on a lack a response to IBMX/FSK. Agonist-induced responses were quantified by double normalising the averaged responses from each well as a fractional change from baseline and then as a percentage of the response to IBMX/FSK. Where indicated, data were plotted using 3-parameter logistic fitting to establish relative potency and maximal responses for statistical analysis.

## cAMP/PKA/ERK imaging using FRET sensors

Plasmid-encoded ^T^Epac^VV^, AKAR4 and EKAR4 biosensors were transfected into wild-type INS-1 832/3 cells using Lipofectamine 2000, with co-transduction of cADDis sensors in some cases as above. For experiments using dispersed islet cells from *Pdx1-CreERT2-CAMPER* mice, 4-OHT was added to the islet culture media after isolation to induce ^T^Epac^VV^ expression *in vitro*; *Sst-Cre-CAMPER* mice do not require 4-OHT to be added. Experiments were then performed as described in Section 4.18, with the exception that an image splitter (Optosplit III, Cairn Research Ltd, UK) was used to simultaneously project CFP and YFP emission images onto separate areas of the camera chip. In some cases, the third (mCherry) channel was used to also acquire responses from an additional sensor. YFP/CFP FRET ratios were calculated per-cell after flat-field correction with background subtraction and cellular responses analysed as in Section 4.18.

## Widefield imaging of total fluorescent agonist binding in intact pancreatic islets

Islets from wild-type mice were treated in complete RPMI-1640 medium with 1 nM ExF1-TMR or ExD3-TMR for 60 minutes continuously, or for 10 minutes followed by transfer to fresh complete RPMI-1640 containing 20 µm exendin(9-39) for 50 minutes. Islets were then washed in PBS prior to imaging using the same microscope as in Section 4.6 by widefield epifluorescence. After flat-field correction, islets (~30-50 per condition and experimental replicate) were manual segmented and TMR fluorescence quantified using Fiji v1.54f.

## Kinome assay

INS-1 832/3 cells were seeded into 12-well plates 24 hours before the assay in complete RPMI-1640 media with 10% FBS and 11 mM glucose. Cells were washed once and agonist (100 nM) or vehicle was added in complete media. For “continuous treatment” wells, the stimulation continued for 60 minutes. For “washout” wells, agonist was removed after 10 minutes and replaced with either complete media, or complete media containing 20 µM exendin(9-39), for a further 50 minutes. The assay was performed at 37°C. At the end of the stimulation period, plates were transferred to the cold room, media was promptly removed, cells were washed with cold PBS, and plates were then stored at -80°C until analysis. Plates were shipped to the central PamGene facility for analysis. Briefly, cell lysates were applied to STK PamChip arrays in a PamStation device, which detects phosphorylation of a panel of target peptide substrates in real time through fluorescence changes. A standard QC and batch-effect correction pipeline was applied to determine phosphorylation signal for each peptide substrate. Agonist-mediated effects were then calculated as log_2_ fold changes relative to the vehicle: for the “continuous” and “non-antagonist washout” groups, vehicles were pooled from all 8 samples, whereas for the “exendin(9-39)-mediated washout” groups, the exendin(9-39)-only “vehicle” was used. ANOVA testing, with Dunnett’s correction was performed to identify statistically significantly increased changes. Upstream kinase analysis (UKA) was applied to phosphosite data to predict kinase activity using a functional class scoring method based on public databases of kinase-protein-phosphosite relationships. UKA was used to ascribe both a “kinase statistic”, which represents the direction and magnitude of all phosphosites engaged by that kinase scaled by the noise, and a “kinase score”, which represents the significance of the change and its specificity for that particular kinase.

## Perfused pancreas study

Isolated pancreas perfusions were performed in male Wistar rats (Janvier, Saint Berthevin Cedex, France), weighing approximately 300 g after at least a week of acclimatisation. As previously described [16,17], isolated rat pancreas perfusion was achieved by, under deep anaesthesia, performing a laparotomy, followed by surgical removal of the small intestine distally to the duodenum, large intestine, stomach, and spleen. The kidneys were also vascularly excluded. Then, the thoracic aorta was closed, and, immediately after, an arterial catheter in the abdominal aorta (perfusion buffer input) and of a venous catheter placed in the pre-hepatic portion of the portal vein (output) were placed. Once flow was established, the rat was euthanized by diaphragmatic perforation, and the organ perfusion setup was stabilized for 30 minutes before any sampling was collected. The flow rate was maintained at 5 mL/min, with Krebs-Ringer perfusion buffer, supplemented with 6 mmol/L glucose, and 5 mmol/L pyruvate, enriched with 95% O_2_ and 5% CO_2_, pH adjusted to 7.4-7.5 and pre-warmed to 37^o^C. To estimate the ability of ExD3 and ExF1 to maintain hormone secretion after washout, AUC was calculated from a period before and after the 10-minute agonist perfusion period for each preparation, with normalisation to the period duration. 10 mmol/L L-arginine was administered at the end of each experiment, as a positive control. The hormonal output was measured using in-house radioimmunoassays (antibodies 2006 and 1758, for insulin and somatostatin, respectively).

## Statistics

Quantitative data were analysed using Prism 10.4.0 (GraphPad Software). In cell culture experiments, technical replicates were averaged so that each individual experiment was treated as one biological replicate. Dose responses were analysed using 3-parameter logistic fits, with constraints imposed as appropriate. Statistical comparisons were made by t-test or ANOVA as appropriate, with paired or matched designs used depending on the experimental design. Mean ± standard error of mean (SEM) or individual replicates are displayed throughout. Statistical significance was inferred if p<0.05.

# Supplementary references

[1] Ast, J., Arvaniti, A., Fine, N.H.F., Nasteska, D., Ashford, F.B., Stamataki, Z., et al., 2020. Super-resolution microscopy compatible fluorescent probes reveal endogenous glucagon-like peptide-1 receptor distribution and dynamics. Nature Communications 11(1): 467, Doi: 10.1038/s41467-020-14309-w.

[2] Depry, C., Allen, M.D., Zhang, J., 2011. Visualization of PKA activity in plasma membrane microdomains. Molecular BioSystems 7(1): 52–8, Doi: 10.1039/c0mb00079e.

[3] Sample, V., DiPilato, L.M., Yang, J.H., Ni, Q., Saucerman, J.J., Zhang, J., 2012. Regulation of nuclear PKA revealed by spatiotemporal manipulation of cyclic AMP. Nature Chemical Biology 8(4): 375–82, Doi: 10.1038/nchembio.799.

[4] Keyes, J., Ganesan, A., Molinar-Inglis, O., Hamidzadeh, A., Zhang, J., Ling, M., et al., 2020. Signaling diversity enabled by Rap1-regulated plasma membrane ERK with distinct temporal dynamics. ELife 9: e57410, Doi: 10.7554/elife.57410.

[5] Klarenbeek, J.B., Goedhart, J., Hink, M.A., Gadella, T.W.J., Jalink, K., 2011. A mTurquoise-based cAMP sensor for both FLIM and ratiometric read-out has improved dynamic range. PloS One 6(4): e19170, Doi: 10.1371/journal.pone.0019170.

[6] Xu, W., Qadir, M.M.F., Nasteska, D., Sa, P.M. de., Gorvin, C.M., Blandino-Rosano, M., et al., 2023. Architecture of androgen receptor pathways amplifying glucagon-like peptide-1 insulinotropic action in male pancreatic β cells. Cell Reports 42(5): 112529, Doi: 10.1016/j.celrep.2023.112529.

[7] Hohmeier, H.E., Mulder, H., Chen, G., Henkel-Rieger, R., Prentki, M., Newgard, C.B., 2000. Isolation of INS-1-derived cell lines with robust ATP-sensitive K+ channel-dependent and -independent glucose-stimulated insulin secretion. Diabetes 49(3): 424 430.

[8] Naylor, J., Suckow, A.T., Seth, A., Baker, D.J., Sermadiras, I., Ravn, P., et al., 2016. Use of CRISPR/Cas9-engineered INS-1 pancreatic  cells to define the pharmacology of dual GIPR/GLP-1R agonists. Biochemical Journal 473(18): 2881–91, Doi: 10.1042/bcj20160476.

[9] Ast, J., Nasteska, D., Fine, N.H.F., Nieves, D.J., Koszegi, Z., Lanoiselée, Y., et al., 2023. Revealing the tissue-level complexity of endogenous glucagon-like peptide-1 receptor expression and signaling. Nature Communications 14(1): 301, Doi: 10.1038/s41467-022-35716-1.

[10] Jun, L.S., Showalter, A.D., Ali, N., Dai, F., Ma, W., Coskun, T., et al., 2014. A Novel Humanized GLP-1 Receptor Model Enables Both Affinity Purification and Cre-LoxP Deletion of the Receptor. PLoS ONE 9(4): e93746, Doi: 10.1371/journal.pone.0093746.

[11] Muntean, B.S., Zucca, S., MacMullen, C.M., Dao, M.T., Johnston, C., Iwamoto, H., et al., 2018. Interrogating the Spatiotemporal Landscape of Neuromodulatory GPCR Signaling by Real-Time Imaging of cAMP in Intact Neurons and Circuits. Cell Reports 22(1): 255–68, Doi: 10.1016/j.celrep.2017.12.022.

[12] Gu, G., Dubauskaite, J., Melton, D.A., 2002. Direct evidence for the pancreatic lineage: NGN3+ cells are islet progenitors and are distinct from duct progenitors. Development (Cambridge, England) 129(10): 2447 2457.

[13] Taniguchi, H., He, M., Wu, P., Kim, S., Paik, R., Sugino, K., et al., 2011. A Resource of Cre Driver Lines for Genetic Targeting of GABAergic Neurons in Cerebral Cortex. Neuron 71(6): 995–1013, Doi: 10.1016/j.neuron.2011.07.026.

[14] Jaccard, N., Griffin, L.D., Keser, A., Macown, R.J., Super, A., Veraitch, F.S., et al., 2014. Automated method for the rapid and precise estimation of adherent cell culture characteristics from phase contrast microscopy images. Biotechnology and Bioengineering 111(3): 504–17, Doi: 10.1002/bit.25115.

[15] Birke, R., Ast, J., Roosen, D.A., Lee, J., Roßmann, K., Huhn, C., et al., 2022. Sulfonated red and far-red rhodamines to visualize SNAP- and Halo-tagged cell surface proteins. Organic & Biomolecular Chemistry 20(30): 5967–80, Doi: 10.1039/d1ob02216d.

[16] Svendsen, B., Larsen, O., Gabe, M.B.N., Christiansen, C.B., Rosenkilde, M.M., Drucker, D.J., et al., 2018. Insulin Secretion Depends on Intra-islet Glucagon Signaling. Cell Reports 25(5): 1127-1134.e2, Doi: 10.1016/j.celrep.2018.10.018.

[17] Svendsen, B., Holst, J.J., 2021. Paracrine regulation of somatostatin secretion by insulin and glucagon in mouse pancreatic islets. Diabetologia 64(1): 142–51, Doi: 10.1007/s00125-020-05288-0.
